# Natural products targeting glycolysis in cancer

**DOI:** 10.3389/fphar.2022.1036502

**Published:** 2022-11-01

**Authors:** Yuanyuan Zhao, Louisa S Chard Dunmall, Zhenguo Cheng, Yaohe Wang, Lingling Si

**Affiliations:** ^1^ National Centre for International Research in Cell and Gene Therapy, Sino-British Research Centre for Molecular Oncology, State Key Laboratory of Esophageal Cancer Prevention & Treatment, School of Basic Medical Sciences, Academy of Medical Sciences, Zhengzhou University, Zhengzhou, China; ^2^ Centre for Cancer Biomarkers & Biotherapeutics, Barts Cancer Institute, Queen Mary University of London, London, United Kingdom

**Keywords:** natural product, cancer, glycolytic enzymes, glycolysis signaling pathway, oncogene, glycolysis

## Abstract

Many energy metabolism pathways exist in cancer, including glycolysis, amino acid metabolism, fatty acid oxidation, and mitochondrial respiration. Tumor cells mainly generate energy through glycolysis to maintain growth and biosynthesis of tumor cells under aerobic conditions. Natural products regulate many steps in glycolysis and targeting glycolysis using natural products is a promising approach to cancer treatment. In this review, we exemplify the relationship between glycolysis and tumors, demonstrate the natural products that have been discovered to target glycolysis for cancer treatment and clarify the mechanisms involved in their actions. Natural products, such as resveratrol mostly found in red grape skin, licochalcone A derived from root of Glycyrrhiza inflate, and brusatol found in Brucea javanica and Brucea mollis, largely derived from plant or animal material, can affect glycolysis pathways in cancer by targeting glycolytic enzymes and related proteins, oncogenes, and numerous glycolytic signal proteins. Knowledge of how natural products regulate aerobic glycolysis will help illuminate the mechanisms by which these products can be used as therapeutics to inhibit cancer cell growth and regulate cellular metabolism.

**Systematic Review Registration**: https://pubmed.ncbi.nlm.nih.gov/, https://clinicaltrials.gov/, http://lib.zzu.edu.cn/

## 1 Introduction

In the 1920s, Warburg and his colleagues discovered the Warburg effect, in which cancer cells can undergo glycolysis in both aerobic and hypoxic environments (Koppenol et al., 2011). On one hand, glycolysis provides sufficient energy and abundant biosynthetic intermediates, such as lipids, amino acids, and nucleic acids, for biosynthesis and energy requirements, which lays the material foundation for growth and development of cancer cells (Wang S. et al., 2021). On the other hand, lactic acid, the end product of glycolysis, damages tumor-infiltrating T cells and NK cells and activates immune suppressive cells, which forms a microenvironment conducive to tumor growth and promotes tumor proliferation and metastasis (Gao et al., 2022).

**FIGURE 1:**
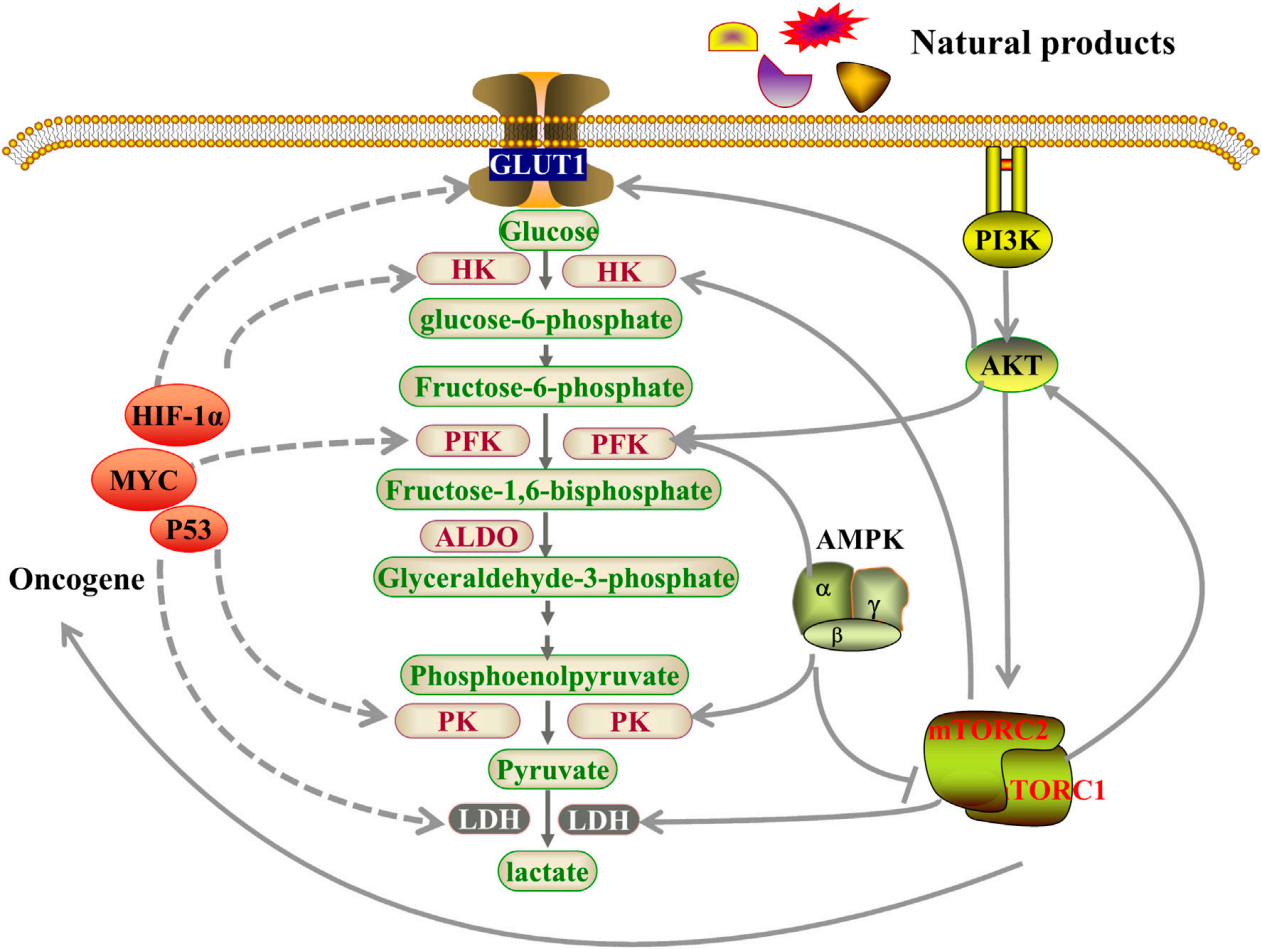
Natural products regulated key factors and signaling pathways that are involved in aerobic glycolysis. Natural products may regulate aerobic glycolysis through three pathways. Firstly, natural products affect glycolysis in tumor cells by directly regulating glycolytic enzymes; Secondly, natural products may regulate genes related to aerobic glycolysis by regulating oncogenes, including HIF-1α, MYC and p53, resulting in changes in the metabolic pathways of tumor cells; Thirdly, natural products can act through the PI3K-AKT-mTOR or AMPK pathways to inhibits tumor cell glycolysis. GLUT1, glucose transporter 1; HK, hexokinase; PFK, Phosphofructokinase; ALDO, aldolase, Fructose-bisphosphate; PK, pyruvate kinase; LDH, lactate dehydrogenase; HIF-1α, Hypoxia Inducible Factor 1 Subunit Alpha; AMPK, AMP-dependent protein kinase; PI3K, Phosphatidylinositol 3-kinase; AKT, protein kinase B; mTOR, mammalian target of rapamycin.

The traditional methods of tumor treatment, such as chemotherapy, radiotherapy, surgery and immunotherapy bring high financial burdens and impose mental and physical stress on patients. Cancer researchers have been studying energy metabolism pathways in cancer cells with an aim to block the source of essential nutrients supporting growth and proliferation of cancer cells. Natural products can be safe and effective for the treatment of tumors and can serve specific biological functions through optimization of their structure (Atanasov et al., 2021). Natural products can inhibit the process of glycolysis and disrupt tumor proliferation and migration by targeting the glycolytic/metabolic phenotype (Figure 1). For example, resveratrol, a polyphenol found in grapes, inhibits glycolysis by activating AMP-activated protein kinase (AMPK), thereby inhibiting colon cancer invasion and migration (Saunier et al., 2017). Natural products targeting glycolysis can also enhance sensitivity of tumor cells to drugs. Several years ago, reports clarified the specific advantages of natural products targeting aerobic glycolysis for the treatment of cancer and their biochemical targets (Wang et al., 2012; Gao and Chen, 2015). In this review, we describe the important factors and related mechanisms that affect glycolysis in tumor cells and classify natural products according to how they regulate glycolytic enzymes and related proteins, oncogenes and glycolytic signaling pathways. We address the implication of key enzymes of the aerobic glycolytic pathway including glucose transporters (GLUTs), hexokinase (HK), phosphofructokinase (PFK) and pyruvate kinase (PK), along with related signaling pathways including protein kinase B/mammalian target of rapamycin pathway (PI3K/AKT/mTOR), adenosine monophosphate-activated protein kinase (AMPK) and oncogenes (HIF-1, c-MYC, and p53), and other latest targets including sirtuin 6 (SIRT6), heat shock protein 90α (HSP90α), glyceraldehyde-3-phosphate dehydrogenase (GAPDH), S-phase kinase-associated protein 2 (Skp2), integrin subunit beta 2 (ITGB2)/focal adhesion kinases (FAKs), microRNAs (miR-491-5p/miR-145/miRNA-34a), tet methylcytosine dioxygenase 3 (TET3) and CD147 in tumor cells.

## 2 Natural products targeting glycolysis in cancer

Natural products are compounds extracted and optimized from nature and can be obtained from plants, microorganisms, animals, insects, minerals, marine organisms, and so forth, which have chemical, functional, and structural diversity (Ji et al., 2009). Natural products derived from traditional Chinese medicine have been used in cancer treatment for centuries and are known for their multi-target pharmacological effects and reduced side effects (Wang S. et al., 2021). Approximately 30% of top-selling drugs are natural products or their derivatives (Newman and Cragg, 2007). More than 60% of anticancer drugs currently in clinical use have natural product sources (Newman and Cragg, 2012).

### 2.1 The classic pathways of natural products targeting glycolysis

#### 2.1.1 Targeting glycolytic enzymes

Glycolytic enzymes play a significant role in tumor progression. The glucose transporter type 1 (GLUT1), encoded by Solute Carrier Family 2 Member 1 (SLC2A1), belongs to the sugar transporter subfamily of the major facilitator superfamily (Holman, 2020) and mediates cellular uptake of glucose into a variety of tissues at basal levels. Cancer cells require an enhanced supply of glucose due to the Warburg effect, leading to an increase in glucose transport in cancer cells, mainly due to the upregulation of GLUT1. The over-activation and high expression of glycolysis-related enzymes, mainly including hexokinase (HK), phosphofructokinase (PFK), pyruvate kinase (PK) and LDHA, is one of the reasons for enhanced aerobic glycolysis in cancer cells. Among these enzymes, HK and PFK1 are two rate-limiting enzymes of glycolysis. HK regulates the total glucose flux that is shunted into two pathways; glycolysis and the pentose phosphate pathway. PFK1 determines the rate at which glucose enters glycolysis. 6-Phosphofructo-2-Kinase/Fructose-2,6-Biphosphatase 2 (PFKFB2) is an enzyme that catalyzes the synthesis of fructose-2,6-bisphosphate during glycolysis. Lactate dehydrogenase (LDH) enzyme catalyzes the reversible conversion of pyruvate to lactate using NADH. LDHA, the most predominant isoforms of LDH, is commonly overexpressed in cancer cells and with higher affinity for pyruvate, leading to an excessive accumulation of lactate, promoting its secretion by the monocarboxylate transporters and increasing the acidification of the tumor microenvironment. In the case of LDHB, with higher affinity for lactate, its upregulation is considered one of the hallmarks of cancer (Beltinger, 2019).

**FIGURE 2:**
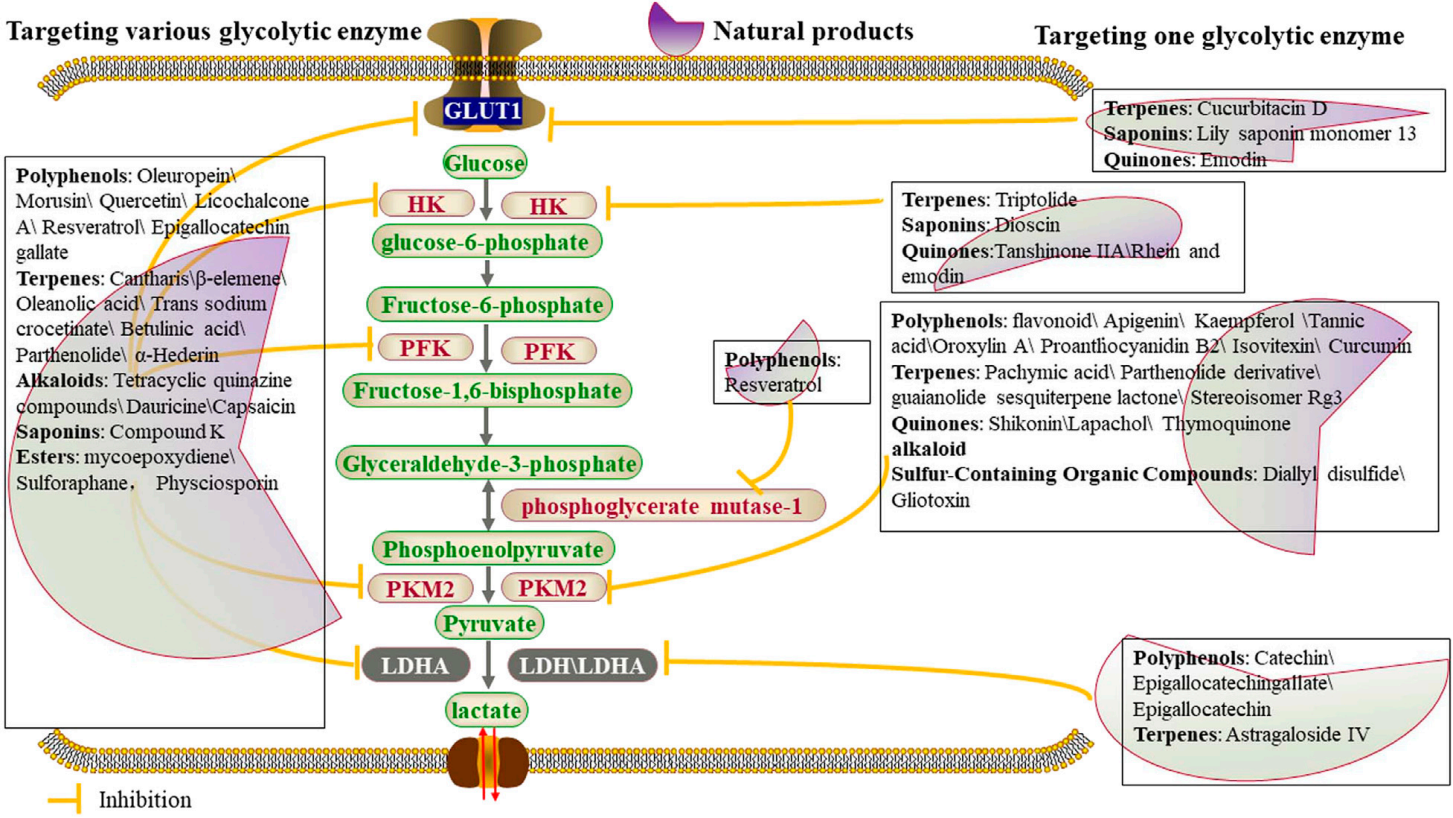
Natural products affect glycolysis in tumor cells by directly regulating glycolytic enzymes.

Numerous natural compounds affect expression of glucose transporters indirectly (Figure 2; Table 1). For example, the isoflavonoids, genistein and quercetin, inhibit aerobic glycolysis by regulating GLUT1. Quercetin, found in many plants and foods, such as red wine, apples, onions, green tea, et al., successfully blocked cell glycolysis by inhibiting the level of glucose uptake and the production of lactic acid, and also decreased the level of glycolysis-related proteins GLUT1, PKM2 and LDH, which further suppressed the progression of breast cancer by inhibiting cell mobility through AKT-mTOR pathway-mediated autophagy induction (Jia et al., 2018). α-Hederin inhibits cell growth *via* activating SIRT6 expression and inhibiting glycolysis and glycolysis related protein expression of GLUT1, HK2, PKM2 and LDHA in A549 lung carcinoma cell lines (Fang et al., 2021a). Cucurbitacin is a tetracyclic triterpenoid that belongs to the cucurbitaceae family and can be isolated from members of the family Cucurbitaceae, such as cucumber (Cucumis sativus) and melon (Cucumis melo L.). Cucurbitacin D treatment suppressed GLUT1 expression by restoring miR-132 in prostate cancer cells and showed potent anticancer activity (Sikander et al., 2019). An olive leaf extract enriched in Oleuropein decreased melanoma cell proliferation and motility and reduced the rate of glycolysis of human melanoma cells without affecting oxidative phosphorylation, which was associated with a significant decrease of GLUT1 and HK2. As revealed in Table 1 **,** many natural products inhibit glycolysis by controlling dysregulated glycolytic enzymes, thereby reducing tumorigenesis and progression. For example, resveratrol and oleanolic acid regulate aerobic glycolysis by targeting HK2 and PFK1. Cinnamon bark is one of the most popular spices obtained from the inner bark of several tree species from the genus *Cinnamomum*. Cinnamon bark extract suppresses metastatic dissemination of MDA-MB-231 human breast cancer cells through decreasing expression of HK2 (Nakayama et al., 2022). Oleanolic acid (OA) is a triterpenoid component widely found in the plants of Oleaceae family. OA blocks glycolysis in gastric cancer cells by reducing the HK2 and PFK1 expression and intracellular activity that was mediated by HIF-1α (Li et al., 2019). Isovitexin, a flavone that was found in an A. annua tea infusion, inhibits cell proliferation and glucose metabolism by downregulation of expression of PKM2 to enhance the antitumor activity of cisplatin against lung cancer cells and improves cisplatin-induced immunotoxicity in mice (Chen R. L. et al., 2021). Green tea behaves as an anti-oxidant and shows anti-tumor effects. Interestingly, matcha green tea inhibits the propagation of cancer stem cells by regulating the expressions of enzyme glycolysis PFKL and PFKP involved in the initial preparatory phase of glycolysis (Bonuccelli et al., 2018). Catechin is a phenolic antioxidant found in chocolate, red wine, green tea, fruits (apricots or cherry), and vegetables including broad beans, and it re-sensitizes gastric cancer cell line SNU620 to 5-fluorouracil by suppressing LDHA activity through binding the substrate-binding site of LDHA and reducing lactate production (Han et al., 2021). In addition, shikonin, a quinone compound present in alkanet roots with a wide spectrum of biologic properties, inhibited tumor growth by suppressing tumor cell aerobic glycolysis in a PKM2-dependent manner in B16 cells (Zhao et al., 2018). Curcumin, isolated from the root of curcuma longa, is the main component of turmeric and also effectively inhibits the proliferation of liver cancer cells by suppressing glycolysis through down-regulating the expression of LDHA (Man et al., 2020). Dauricine, the major bioactive component isolated from the roots of Menispermum dauricum D.C, has shown promising pharmacological activities with a great potential for clinic use, which inhibited glucose glycolysis and increased oxidative phosphorylation by downregulating the expression of HK2 and PKM2 directly targeted by miR-199a In hepatocellular carcinoma cells (Li W. et al., 2018).

**TABLE 1:** Natural products regulate glycolytic enzymes in cancer.

Ingredients	Target glycolytic enzymes	Adjustment method	Tumor	Source	Category	References
emodin	GLUT1	down-regulate	renal cell carcinoma	rhubarb	anthraquinone	Wang et al. (2021a)
cucurbitacin D	GLUT1	down-regulate	prostate cancer	cucurbitaceae	tetracyclic triterpenoid	Sikander et al. (2019)
Saponin monomer 13 of the dwarf lilyturf tuber	GLUT1	down-regulate	colorectal cancer	dwarf lilyturf tuber	saponin monomer	Wei et al. (2019)
phenethyl isothiocyanate	GLUT1 HK2	down-regulate	prostate cancer	cruciferous vegetables	isothiocyanate	Singh et al. (2018)
genistein	GLUT1, HK2	down-regulate	hepatocellular carcinoma	soy products	isofavonoid	Li et al. (2017)
parthenolide	GLUT1 HXK II	down-regulate	colorectal cancer	extracts of Mexican Indian medicinal plants	sesquiterpene lactone	Kim et al. (2017)
physciosporin	GLUT1, HK2, PKM2	down-regulate	breast cancer	species of genus Pseudocyphellaria	depsidone	Taş et al. (2021)
α-Hederin	GLUT1, HK2, PKM2, LDHA	down-regulate	lung cancer	pulsatilla chinensis	pentacyclic triterpenoid saponin	Fang et al. (2021a)
oleuropein	GLUT1, PKM2	down-regulate	melanoma	olea europaea L	phenolic	Ruzzolini et al. (2020)
cantharis	GLUT1, PKM2	down-regulate	breast cancer	insect	cantharidin	Pan et al. (2019b)
tetracyclic quinazine compounds	GLUT1, PKM2	down-regulate	colorectal cancer	root of sophora flavescens ait	oxymatrine	Li et al. (2020b)
morusin	HK2, PKM2, LDH	down-regulate	hepatocellular carcinoma	the roots of morus alba	flavonoid	Cho et al. (2021)
quercetin	GLUT1, PKM2, LDHA	down-regulate	breast cancer	Leaves, fruits, vegetables	flavonoid	Jia et al. (2018)
β-elemene	GLUT1, PKM2, LDHA	inhibit	breast cancer	curcuma zedoary	terpene	Pan et al. (2019a)
licochalcone A	GLUT1, PDK1	down-regulate	hypoxic cancer	gycyrrhiza uralensis	phenol chalconoid	Park et al. (2021b)
tanshinone IIA	HK2	down-regulate	oral squamous cell carcinoma	salvia miltiorrhiza	diterpenoid naphthoquinone	Li et al. (2020a)
dioscin	HK2	down-regulate	colorectal cancer	plants	steroidal saponin	Zhou et al. (2020)
triptolide	HK2	down-regulate	non-small cell lung cancer	root extracts of tripterygium wilfordii	pentacyclic triterpenoid	Hamdi et al. (2018)
rhein	HK2	inhibit	Liver cancer cell	rheum palmatum	monomeric anthraquinone	Wu et al. (2019)
sulforaphane	HK2, PK	down-regulate	prostate cancer	broccoli extract	isothiocyanate	Carrasco-Pozo et al. (2019)
oleanolic acid	HK2, PFK1	down-regulate	gastric tumor cell	leaves and roots of oleaceae plants	pentacyclic triterpenoid saponin	Li et al. (2019)
compound K	HK2, PKM2	down-regulate	hepatocellular carcinoma	saponin	a metabolite of the ginsenosides	Shin et al. (2021)
dauricine	HK2, PKM2	down-regulate	hepatocellular carcinoma	roots of Menispermm dauricum D.C.	alkaloid	(Jin et al., 2010; Li et al., 2018d)
epigallocatechin gallate	PFK	down-regulate	colorectal cancer	green tea	polyphenol	Chen et al. (2022a)
kaempferol	PKM2	down-regulate	colon cancer	natural foods	polyphenol	Wu et al. (2021)
tannic acid	PKM2	down-regulate	colon cancer	grapes and green tea	polyphenolic acid	Yang et al. (2018)
proanthocyanidin B2	PKM2	down-regulate	hepatocellular carcinoma	grape seed, pine bark, wine, and tea leaves	dimer flavonoid	Feng et al. (2019)
isovitexin	PKM2	down-regulate	non-small cell lung cancer	food byproducts and medicinal plants	flavonoid	Chen et al. (2021a)
pachymic acid	PKM2	down-regulate	breast cancer	poria cocos	triterpenoid	Miao et al. (2019)
Parthenolide derivative	PKM2	down-regulate	glioblastoma	feverfew	germacrane sesquiterpene lactone	Ding et al. (2020)
Shikonin	PKM2	inhibit	bladder cancer	lithospermum erythrorhizon	naphthoquinone analog	Wang et al. (2018)
Curcumin	PKM2	down-regulate	lung cancer	rhizome of the plant curcuma longa	phyto polyphenol	Siddiqui et al. (2018a)
Lapachol	PKM2	down-regulate	melanoma	the bark of tabebuia avellanedae	analog of shikonin	Shankar Babu et al. (2018)
Micheliolide	PKM2	down-regulate	leukemia	michelia champaca plants	guaianolide sesquiterpene lactone	Li et al. (2018c)
diallyl disulfide	PKM2	down-regulate	breast cancer		sulfur-containing organic	Xie et al. (2018)
Gliotoxin	PKM2	down-regulate	glioma	marine-derived fungal secondary metabolite	sulfur-containing organic	Tang et al. (2020)
Shikonin	PKM2	down-regulate	lung carcinoma	lithospermumn erythrorhizon	naphthoquinone	Zhao et al. (2018)
capsaicin	PKM2, LDHA	down-regulate	sepsis	capsicum	isothiocyanate	Zhang et al. (2022a)
Catechin	LDHA	down-regulate	gastric cancer	green tea	polyphenol	Han et al. (2021)
epigallocatechin gallate	LDHA	down-regulate	breast and pancreatic cancer	green tea	polyphenol	Lu et al. (2015)
astragaloside IV	LDHA	down-regulate	gastric carcinoma	astragalus membranaceus	triterpenoid saponin	Zhang et al. (2018)
betulinic acid	LDHA, p-PDK1, PDK1	down-regulate	breast cancer	birch bark	pentacyclic terpene	Jiao et al. (2019a)
Scopolin	PGK2, GPI, GPD2	inhibit	hepatocellular carcinoma	smilax china L	alkaloid	Wang et al. (2022)
cardamonin	PDHK1	down-regulate	breast cancer	alpinia katsumadai	chalcone	Jin et al. (2019a)
Erianin	pyruvate carboxylase	inhibit	cancers	plants of the genus dendrobium	dibenzyl compound	Hong et al. (2022)

#### 2.1.2 PI3K-AKT-mTOR pathway

Phosphatidylinositol 3-kinases (PI3Ks) are a family of signaling enzymes that include three major classes of lipid kinases. Class I PI3Ks generate 3-phosphoinositides in response to growth stimuli. PI3K activates the serine/threonine kinase AKT (also known as protein kinase B or Protein kinase B, PKB) in its downstream signaling pathway, which plays an important role in regulating various cellular functions including metabolism, growth, proliferation, survival, transcription, and protein synthesis (Manning and Toker, 2017). Mammalian target of rapamycin (mTOR) is one of the downstream signals of AKT. AKT increases the translation of the transcription factor HIF-1α by phosphorylating mTORC1, which activates the glycolytic enzyme PFK (Song et al., 2020). AKT can also activate mTOR complex 2 (mTORC2), which is associated with enhanced glycolysis (Huang et al., 2016). The enzyme MTORC2 promotes cell survival, glucose uptake and glycolysis through activating AGC kinase family proteins, including AKT and protein kinase C (PKC) (Hua et al., 2019). Activated PI3K/AKT signaling stimulates glucose uptake and enhances glycolysis and lipid biosynthesis by regulating the expression of GLUT1 (Jin et al., 2021), driving lactate production and inhibiting the degradation of macromolecules in cancer cells, affecting tumor cell metabolism (Wasik and Lehtonen, 2018).

**FIGURE 3:**
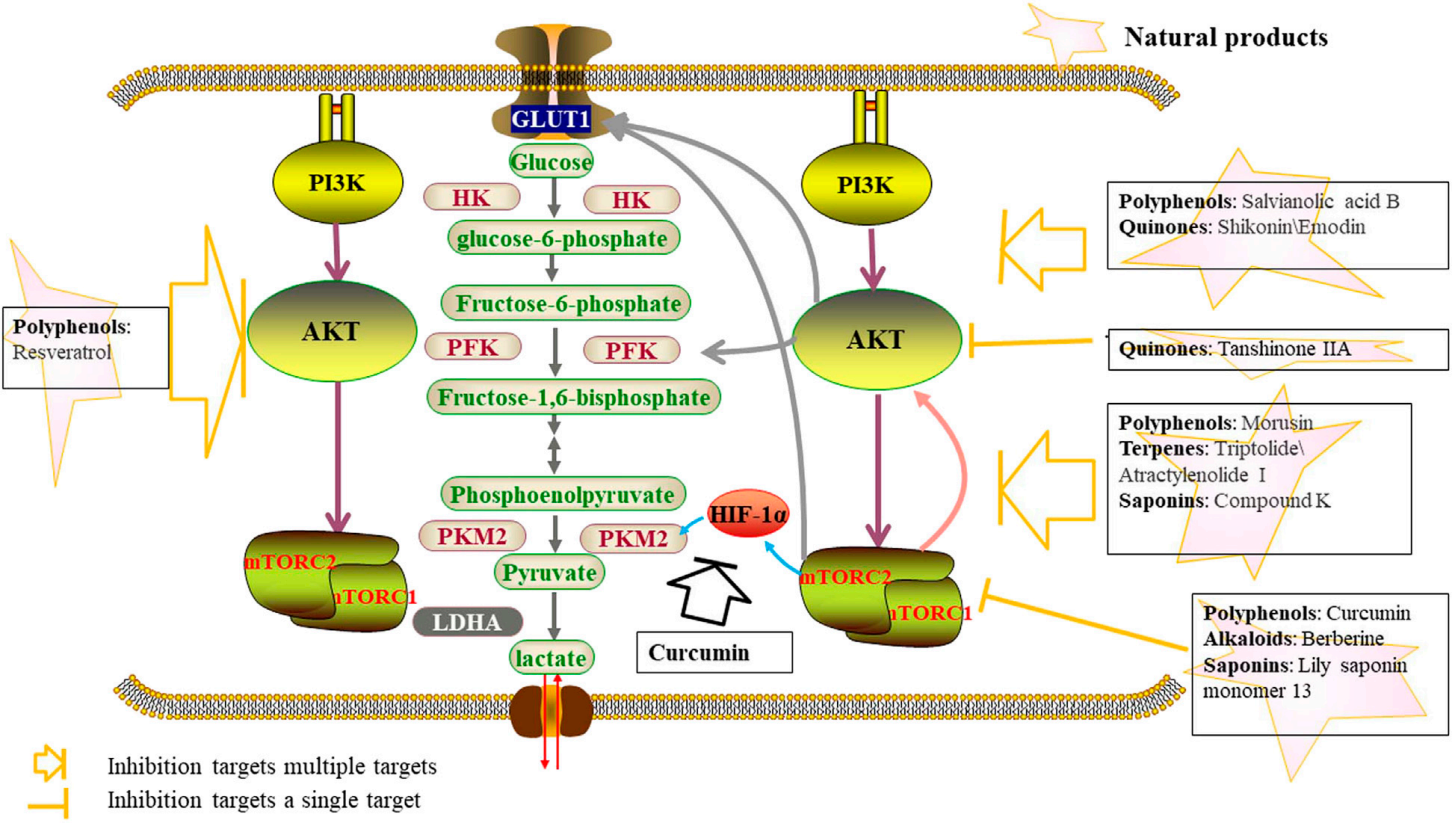
Natural products inhibit glycolysis through the PI3K-AKT-mTOR pathway.

Emodin (1, 3, 8-trihydroxy-6-methylanthraquinone) is a derived anthraquinone compound extracted from the leaves, roots and barks of pharmaceutical plants, including aloe vera, cascara, rhubarb et al., and inhibits glycolysis by downregulation of GLUT1 through ROS-mediated inactivation of the PI3K/AKT signaling pathway (Wang K. J. et al., 2021). Curcumin, the main polyphenol pigment in the plant turmeric with antioxidant properties, down-regulates PKM2 expression by inhibiting the mTOR-HIF1α axis, thereby inhibiting glucose uptake and lactate production in various cancer cell lines (Siddiqui et al., 2018a). The activation of the PI3K/AKT signaling pathway abolished the antitumor effect of a naphthoquinone derivative Shikonin, derived from the root of the herbal plant, which indicated that Shikonin suppressed the progression of nasopharyngeal cancer through inactivation of the PI3K/AKT signaling pathway (Zhang et al., 2020). Atractylenolide 1, an active component of Atractylodes Lancea, down-regulates the phosphorylation of AKT/mTOR pathway-related proteins and effectively inhibited the proliferation and invasion of colorectal cancer cells by acting as an inhibitor of AKT/mTOR (Wang et al., 2020). Tanshinone IIA, a diterpenoid naphthoquinone extracted from Salvia miltiorrhiza, attenuates oral squamous cell carcinoma (OSCC) cells by reducing AKT/c-MYC signaling and enhancing c-MYC ubiquitination and degradation, which results in a reduction in HK2-mediated aerobic glycolysis in OSCC (Li M. et al., 2020) (Figure 3; Table 2).

**TABLE 2:** Natural products regulate glycolysis through the PI3K-AKT-mTOR signaling pathway in cancer.

Ingredients	Target	Adjustment method	Target tumor	Source	Category	References
resveratrol	PI3K, AKT, mTOR	down-regulate	cancers	grapes, berries, peanuts, red wine	polyphenol	Brockmueller et al. (2021a)
salvianolic acid B	PI3K, AKT	down-regulate	oral squamous cell carcinoma	salviae miltiorrhizae	polyphenol	Wei et al. (2018b)
Shikonin	PI3K/AKT	inactivate	nasopharyngeal carcinoma	root of the herbal plant	naphthoquinone	Zhang et al. (2020)
Emodin	PI3K, AKT	down-regulate	renal cell carcinoma	Rhubarb	anthraquinone	Wang et al. (2021a)
triptolide	AKT, mTOR	down-regulate	non-small cell lung cancer	root extracts of tripterygium wilfordii	pentacyclic triterpenoid	Hamdi et al. (2018)
atractylenolide I	AKT, mTOR	down-regulate	colorectal cancer	plant-based baizhu	sesquiterpenoids	Wang et al. (2020)
tanshinone IIA	AKT	down-regulate	oral squamous cell carcinoma	salvia miltiorrhiza	diterpenoid naphthoquinone	Li et al. (2020a)
compound K	AKT, mTOR	down-regulate	hepatocellular carcinoma	Saponin	metabolite of the ginsenoside	Shin et al. (2021)
Morusin	AKT mTOR	down-regulate	hepatocellular carcinoma	the roots of morus alba	flavonoid	Cho et al. (2021)
Berberine	mTOR	down-regulate	colon cancer	roots, rhizomes, stems, and bark of berberis plan	isoquinoline alkaloid	Mao et al. (2018a)
curcumin	mTOR	down-regulate	lung cancer	rhizome of the plant curcuma longa	polyphenol	Siddiqui et al. (2018a)
lily saponin monomer 13	mTOR	down-regulate	colorectal cancer	dwarf lilyturf tuber	saponin monomer	Wei et al. (2019)

#### 2.1.3 The AMPK signaling pathway

**FIGURE 4:**
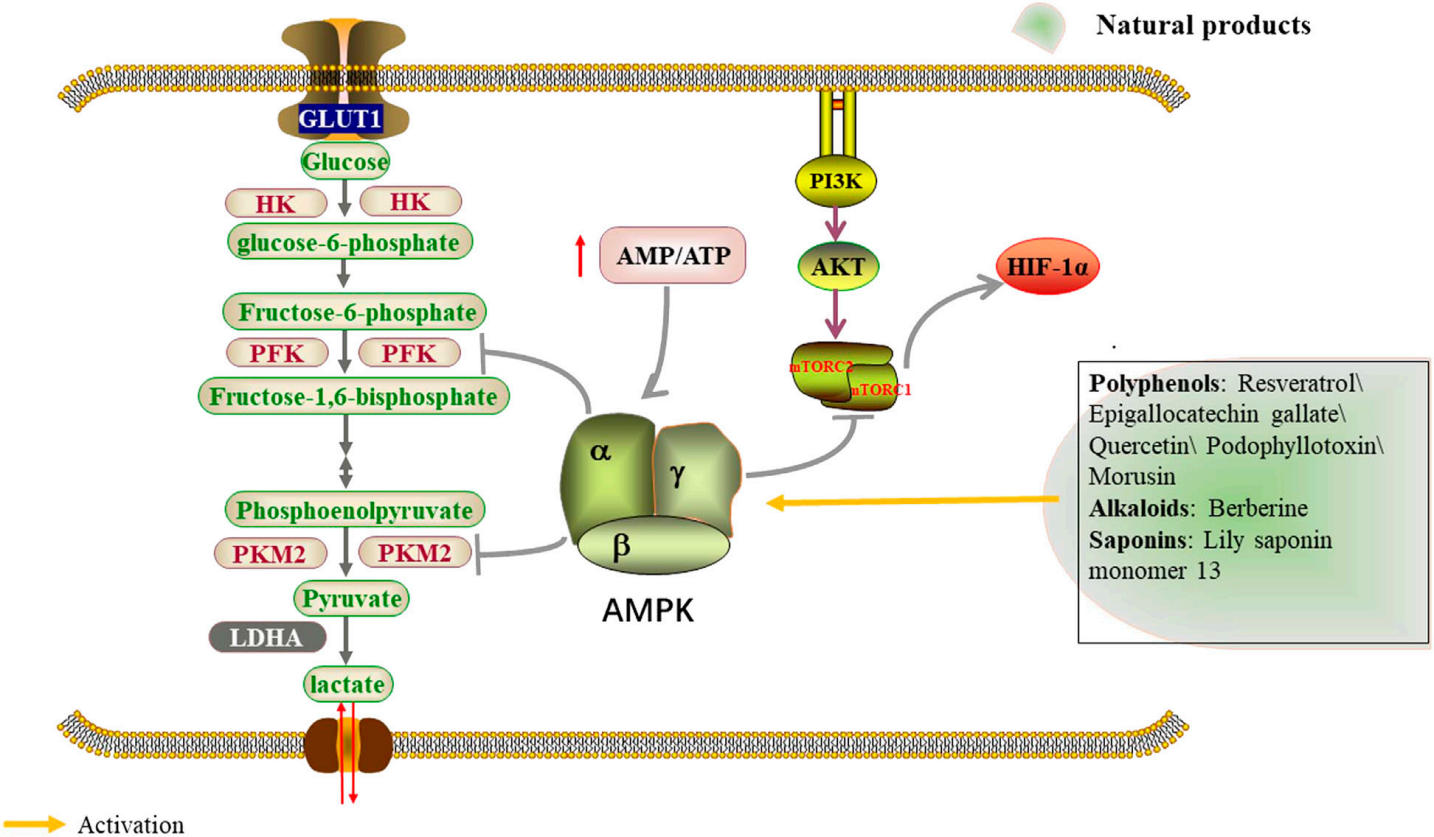
Natural products regulate glycolysis through the AMPK signaling pathway.

AMPK exists as a heterotrimeric complex, consisting of a catalytic *a* subunit and accessory *ß* and *γ* subunits (Steinberg and Carling, 2019). It is activated upon changes in energy availability, and thus changes in the ATP-to-ADP or ATP-to-AMP ratio (Herzig and Shaw, 2018). Once activated, AMPK redirects metabolism towards increased catabolism and decreased anabolism through the phosphorylation of key proteins in multiple pathways, including the mTOR complex 1 (mTORC1) (Benito-Cuesta et al., 2021), glycolysis (Kalezic et al., 2021) and mitochondrial homeostasis (Trefts and Shaw, 2021) pathways. LKB1, an upstream kinase of AMPK, phosphorylates and activates the catalytic subunit of AMPK at its T-loop residue Thr 172 in response to increased AMP/ATP ratios under metabolic stress (Kottakis and Bardeesy, 2012) (Figure 4; Table 3). Podophyllotoxin (PTOX), a well-known naturally aryltetralinlignane extracted from Podophyllum peltatum and a new PTOX derivative compound SU212, exhibited selective anticancer toxicity through direct activation of AMPK, which could regulate glycolysis through the AMPK/HIF-1α pathway in triple-negative breast cancer cells suggesting the potential research interest of PTOX derivatives in the field of tumor glycolysis (Tailor et al., 2021). Lily saponin monomer 13, a saponin monomer derived from lily flower, suppresses colorectal cancer cell proliferation by activating the AMPK pathway and blocking GLUT1 (Wei et al., 2019). Morusin, isolated from the root of morus alba, significantly activated phosphorylation of AMPK/acetyl-CoA carboxylase, but attenuated the expression of the mammalian target of AKT, mTOR, c-MYC, HK2, PKM2, and LDH in Hep3B and Huh7 cells (Cho et al., 2021).

**TABLE 3:** Natural products regulate glycolysis by targeting AMPK in cancer.

Ingredients	Target glycolysis	Adjustment method	Target tumor	Source	Category	References
Morusin	AMPK	up-regulate	hepatocellular carcinoma	the roots of morus alba	flavonoid	Cho et al. (2021)
podophyllotoxin	AMPK	active	cancers	podophyllum peltatum	aryltetralinlignane	Fan et al. (2021)
lily saponin monomer 13	AMPK	active	colorectal cancer	dwarf lilyturf tuber	saponin monomer	Wei et al. (2019)
arsenic trioxide cooperates cryptotanshinone	AMPK	activate	epatocellular carcinoma	cryptotanshinone	salvia miltiorrhiza	Jiang et al. (2022)

#### 2.1.4 Targeting oncogenes

The mutation and abnormal expression of proto-oncogenes creates oncogenes, which achieve acquisition of nutrients through enhancing the activity of glycolysis enzymes in tumor cells and maintain survival and development of cancer cells through the reprogramming of glycolysis metabolism (Mukhopadhyay et al., 2021). It is estimated that increased MYC expression is responsible for at least 40% of human cancers (Wokolorczyk et al., 2008). The link between MYC and regulation of glucose metabolism was first established when an early unbiased screen for MYC target genes uncovered LDHA among 20 other putative MYC target genes, and many other glucose metabolism genes directly regulated by MYC were subsequently documented, including GLUT1, HK2, PFKM, and enolase 1 (Dang et al., 2009). The environment supporting tumor growth is affected by oxygen deficiency (Rani et al., 2022). High expression levels of hypoxia-inducible factor-1a (HIF-1α) and its target genes have been shown to promote tumor aggressiveness. Cancer cells utilize the activated transcription factor HIF-1α to increase glucose uptake and increase glycolytic flux to promote glucose catabolism and adapt to hypoxic environments, ensuring tumor growth (Lee et al., 2020).

**FIGURE 5:**
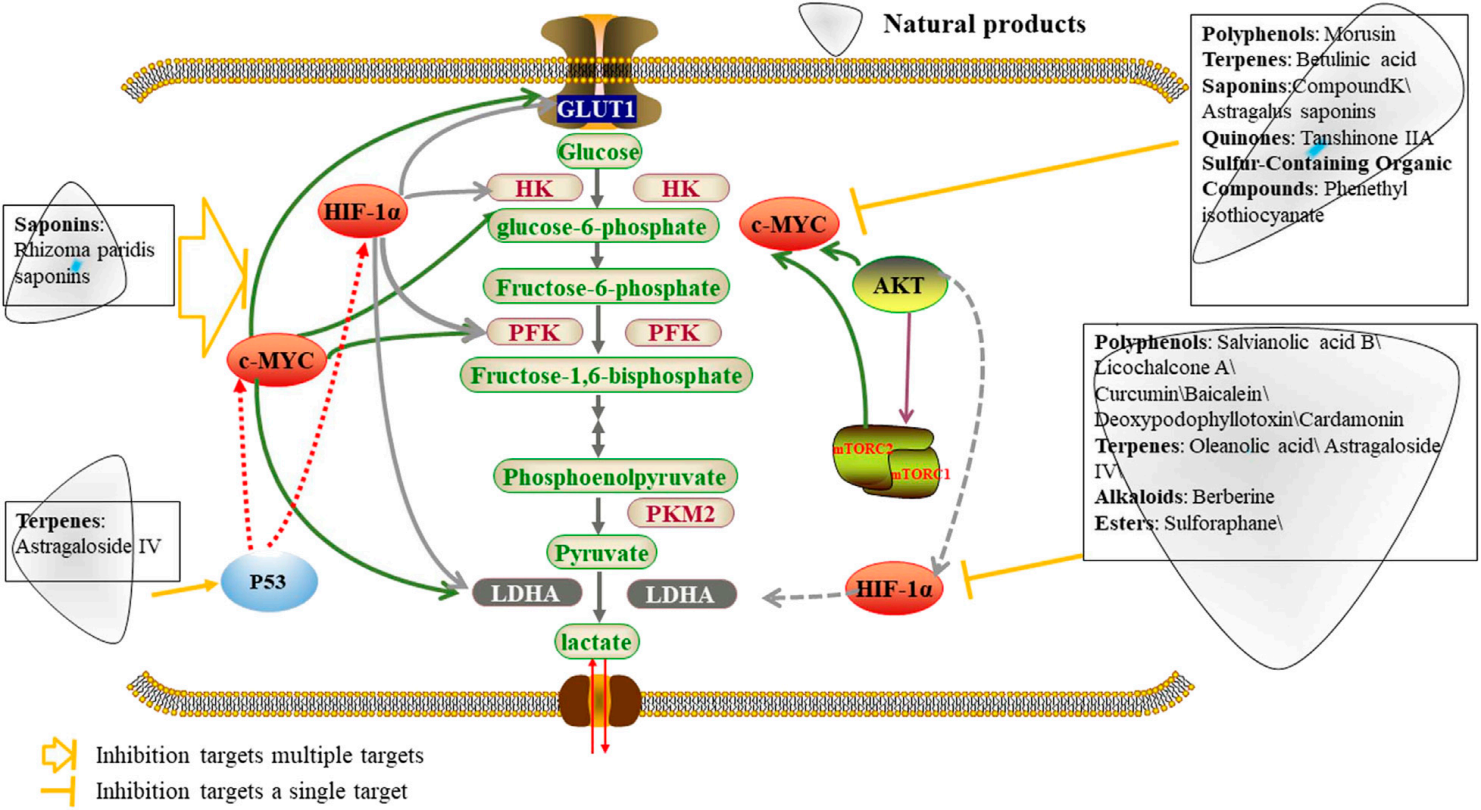
Natural products inhibit aerobic glycolysis by targeting oncogenes.

Accumulating evidence demonstrates that natural products can regulate known oncogenes, including MYC and HIF1-α, that contribute to the genesis of many human cancers, by altering glycolysis to inhibit tumor progression (Figure 5; Table 4). Saponins, including tanshinone IIA, betulinic acid and compound K, were reported to target MYC and inhibit tumor glycolysis in tumors (Jiao et al., 2019a; Li M. et al., 2020; Shin et al., 2021). Tanshinone IIA, isolated from Danshen, decreased glucose consumption, lactate production, and promoted intrinsic apoptosis in oral squamous cell carcinoma cells through inhibition of AKT-c-MYC signaling and promotion of E3 ligase FBW7-mediated c-MYC ubiquitination and degradation, which eventually reduced HK2 expression at the transcriptional level (Li M. et al., 2020). Betulinic acid is a natural pentacyclic triterpenoid that is found in the bark and other plant parts of several species of plants including Syzygium claviflorum. It inhibits aerobic glycolysis activity by hampering lactate production, glucose uptake and extracellular acidification rate, as well as suppressing aerobic glycolysis-related proteins including c-MYC, LDH-A and p-PDK1/PDK1 (pyruvate dehydrogenase kinase 1) in breast cancer cell lines MCF-7 and MDA-MB-231 (Jiao et al., 2019a). Compound K, a ginseng saponin metabolite found in minute quantities of aged ginseng, was shown to induce apoptosis *via* inhibition of glycolysis and AKT/mTOR/c-MYC signaling in 7 human hepatocellular carcinoma cell lines and is a potent anticancer candidate for liver cancer (Shin et al., 2021).

**TABLE 4:** Natural products regulate glycolysis *via* oncogenes in cancer.

Ingredients	Target glycolysis	Adjustment method	Target tumor	Source	Category	References
betulinic acid	c-MYC	down-regulate	breast cancer	birch bark	pentacyclic terpene	Jiao et al. (2019a)
compound K	c-MYC	down-regulate	hepatocellular carcinoma	saponin	a metabolite of the ginsenosides	Shin et al. (2021)
astragalus saponins	c-MYC	down-regulate	colorectal cancer	medicinal herb radix astragali	total saponin	Guo et al. (2019)
tanshinone IIA	c-MYC	down-regulate	oral squamous cell carcinoma	diterpenoid naphthoquinone	salvia miltiorrhiza	Li et al. (2020a)
Morusin	c-MYC	down-regulate	hepatocellular carcinoma	the roots of Morus alba	flavonoid	Cho et al. (2021)
phenethyl isothiocyanate	c-MYC	down-regulate	prostate cancer	cruciferous vegetables	isothiocyanate	Singh et al. (2018)
salvianolic acid B	HIF-1α	down-regulate	oral squamous cell carcinoma	salviae miltiorrhizae	polyphenol	Wei et al. (2018b)
licochalcone A	HIF-1α	down-regulate	hypoxic cancer	glycyrrhiza uralensis	phenol chalconoid	Park et al. (2021b)
curcumin	HIF-1α	down-regulate	lung cancer	rhizome of the plant Curcuma longa	phyto polyphenol	Siddiqui et al. (2018a)
Baicalein	HIF-1α	down-regulate	tamoxifen-resistant breast cancer	Scutellaria baicalensis	Polyphenol	Chen et al. (2021a)
oleanolic acid	HIF-1α	down-regulate	gastric tumor cell	leaves and roots of oleaceae family plants	pentacyclic triterpenoid saponin	Li et al. (2019)
Deoxypodophyllotoxin	HIF-1α	down-regulate	non-small cell lung cancer	anthriscus sylvestris (L.) Hoffm	natural lignans	Yang et al. (2021)
parthenolide	HIF-1 *a*	down-regulate	colorectal cancer	extracts of Mexican Indian medicinal plants	sesquiterpene lactone	Kim et al. (2017)
astragaloside IV	HIF-1α	down-regulate	gastric carcinoma	astragalus membranaceus	triterpenoid saponin	Zhang et al. (2018)
cardamonin	HIF-1α	down-regulate	breast cancer	alpinia katsumadai	chalcone	Jin et al. (2019a)
berberine	HIF-1α	down-regulate	colon cancer	roots rhizomes stems, bark of Berberis plan	isoquinoline alkaloid	Mao et al. (2018a)
resveratrol	HIF-1α/ROS	down-regulate	Cancers	grapes, berries, peanuts, red wine	polyphenol	Brockmueller et al. (2021a)
astragaloside IV	p53	up-regulate	gastric carcinoma	astragalus membranaceus	triterpenoid saponin	Zhang et al. (2018)

In recent years, a growing number of natural products targeting HIF-1α to reduce HIF-1α expression and inhibit tumor glycolysis have been investigated to treat tumors. Polyphenols, including curcumin, salvianolic acid B, licochalcone A and baicalein, have anticancer potential through suppression of the HIF-1α pathway (Wei et al., 2018a; Man et al., 2020; Park et al., 2021a; Chen Y. et al., 2021). Among these components, curcumin, a yellow pigment found primarily in turmeric, enhanced the antitumor effect of sorafenib in hepatocellular carcinoma *via* inhibition of LDH and HIF-1α to suppress aerobic glycolysis (Man et al., 2020). In addition, salvianolic acid B, the most abundant and bioactive water-soluble compound of Salviae miltiorrhizae, has been reported to inhibit aerobic glycolysis as well as PI3K/AKT and HIF-1α signaling pathways in two well-characterized oral squamous cell carcinoma Cal27 and HN4 cell lines (Wei et al., 2018a). Licochalcone A, a chalconoid derived from root of Glycyrrhiza inflate, enhances intracellular oxygen concentations by directly inhibiting mitochondrial respiration, resulting in oxygen-dependent HIF-1α degradation (Park et al., 2021a). Terpenoids, such as oleanolic acid, parthenolide and astragaloside IV, exert an anti-cancer effect by regulating glycolysis and HIF1α expression (Li et al., 2019). Oleanolic acid (OA), a common triterpenoid, is abundantly present in the family Oleaceae, including Olea europaea (olive), other dietary. OA induced HIF-1α-mediated aerobic glycolysis and proliferation by decreasing the expression and intracellular activities of glycolysis rate-limiting enzymes HK2 and PFK1 and downregulating HIF-1α expression in human gastric tumor cells (Li et al., 2019). Curcumin is a polyphenolic yellow spice derived from the rhizomes of Curcuma longa L. plant. It can suppress the two key glycolysis-regulating proteins including hypoxia-inducible factor 1-alpha (HIF-1α) and pyruvate dehydrogenase kinase 1 (PDK1) and target and cellular metabolism by promoting the differential expression of let-7C in ACHN human kidney cancer cells (Obaidi et al., 2022). Cardamonin, a chalcone isolated from Alpiniae katsumadai, reduced glucose uptake as well as lactic acid production and efflux and inhibits breast cancer growth by repressing HIF-1α-dependent metabolic reprogramming (Jin et al., 2019a).

#### 2.1.5 Targeting other signalling pathway to regulate glycolysis

Natural products exert antitumor activity by increasing antioxidant agents, induction of apoptotic factors, modulation of the immune system and decreasing glucose uptake and lactate production. Accordingly, the main targets of natural products includes SIRT6, glycerol-3-phosphate dehydrogenase (GPD2), Hsp90α, GAPDH, S-phase kinase-associated protein 2 (Skp2), ITGB2/focal adhesion kinases (FAKs), miR-491-5p/PKM2, miR-145, p53/miRNA-34a/LDHA, p53/TP53-induced glycolysis and apoptosis regulator (TIGAR), tet methylcytosine dioxygenase 3 (TET3), CD147, lncRNA SNHG10/miR-1271-5p/TRIM66 and pyruvate dehydrogenase kinase 1 (PDK1) (Table 5). Natural compounds can act as modulators of SIRT6, an NAD + dependent histone deacetylase enzyme and a unique Sirtuin family member in treatment of cance r (Akter et al., 2021). *a*-Hederin, a potent bioactive compound of *Pulsatilla chinensis* (Bunge) Regel (Ranunculaceae), inhibited glucose uptake and ATP generation; and reduced lactate production by activating SIRT6 in A549 cells (Fang et al., 2021a). Scopolin obtained from *Smilax china* L. plays the role in controlling hepatocellular carcinoma by regulating the expression of glycolysis proteins glucose-6-phosphate isomerase (GPI), GPD2, mitochondrial and phosphoglycerate kinase 2 (PGK2) and affecting the interaction between Hsp90α and GPD2, which may provide a novel potential treatment direction for hepatocellular carcinoma (Wang et al., 2022). GAPDH exerts metabolic flux control during aerobic glycolysis and therefore is an attractive therapeutic target for cancer. 1,2,3,4,6-penta-O-galloyl-β-D-glucopyranose (PGG) downregulated the GAPDH-dependent glycolysis pathway in LPS-stimulated macrophages, which represents a novel class of GAPDH inhibitors (Li et al., 2022a). Parthenolide was identified as a moderate activator of PKM2, a vital kinase in the glycolysis system, and showed significant anti-Glioblastoma multiforme activity and significantly suppressed tumor growth in the HT29 xenograft model (Ding et al., 2020; Liu X. et al., 2021). Dioscin, a natural steroid saponin derived from several plants, significantly inhibited glycolysis in colorectal cancer cells growth through regulating Cdh1-mediated F-box protein Skp2 degradation (Zhou et al., 2020). Bufalin is extracted from traditional Chinese medicine Chansu and was reported to inhibit cellular glycolysis-induced cell growth and proliferation through repression of the ITGB2/FAKs pathway in ovarian cancer cells (Li H. et al., 2018). MicroRNAs are responsible for the regulation of the key enzymes in glycolysis. Berberine is an alkaloid extracted from coptis, phellodendron and three needles. It increased TET3-mediated demethylation and promoted the suppression of miR-145 on HK2 to antagonize the Warburg effect of ovarian cancer cells (Li et al., 2021). Asragaloside IV (ASIV), one of active compounds in *A. membranaceus*, can inhibit glycolysis through dually mediating p53/miRNA-34a/LDHA and p53/TIGAR pathways and also suppresses glycolic processes *via* restoring aberrance of Monocarboxylate transporter 1/4 (MCT1/4), CD147, and HIF-1α (Zhang et al., 2018). LncRNA SNHG10 promoted glucose uptake *via* interacting with related miRNA in osteosarcoma and up-regulated TRIM66 *via* sponging miR-1271-5p (He et al., 2020). The Chinese medicine Qi Ling inhibited docetaxel resistance and glycolysis of castration-resistant prostate cancer possibly *via* lncRNA SNHG10/miR-1271-5p/TRIM66 pathway (Cao et al., 2021). Oviductus ranae, the dried oviduct of mature female Rana dybowskii, is a famous traditional animal-based medicine, which inhibits the growth, metastasis and glycolysis of hepatocellular carcinoma cells by targeting miR-491-5p/PKM2 axis (Xu et al., 2018).

**TABLE 5:** Natural products regulate glycolysis through the lasted signal pathway in cancer treatment.

Ingredients	Target glycolysis	Adjustment method	Target tumor	Source	Category	References
α-Hederin	SIRT6	active	lung cancer	pulsatilla chinensis	pentacyclic triterpenoid saponin	Fang et al. (2021a)
scopolin	PGK2 GPI GPD2	inhibit	hepatocellular carcinoma	smilax china L	alkaloid	Wang et al. (2022)
1,2,3,4,6-penta-O-galloyl-β-D-glucopyranose	GAPDH	inhibit	glioblastoma multiforme	plants	a tannin family compound	Ding et al. (2020)
parthenolide	PKM2	active	colorectal cancer	extracts of Mexican Indian medicinal plants	sesquiterpene lactone	Liu et al. (2021b)
Dioscin	Skp2	down-regulate	colorectal cancer	plants	steroidal saponin	Zhou et al. (2020)
Bufalin	ITGB2/FAKs pathway	inhibit	ovarian cancer	traditional Chinese medicine Chansu	major digoxin-like component	Li et al. (2018d)
berberine	miR-145	up-regulate	colon cancer	roots rhizomes stems, bark of Berberis plan	isoquinoline alkaloid	Li et al. (2021)
Astragaloside IV	MCT1 MCT4 CD147/miRNA-34a TIGAR	down-regulate	gastric carcinoma	A.membranaceus	a marker for the active constituent	Zhang et al. (2018)
oviductus ranae	miR-491-5p PKM2	down-regulate	hepatocellular carcinoma	dried oviduct of mature female Rana dybowskii	traditional animal-based medicine	Xu et al. (2018)
Curcumin	GLUT 1 PKM LDHA AKT	down-regulate	liver cancer	rhizome of the plant curcuma longa	phyto polyphenol	Man et al. (2020)
Curcumin	P53	up-regulate	liver cancer	rhizome of the plant curcuma longa	phyto polyphenol	Man et al. (2020)
Resveratrol	PKM2	down-regulate	melanoma	grapes, berries, peanuts, red wine	polyphenol	Jia et al. (2021)
Resveratrol	AMPK	up-regulate	melanoma	grapes, berries, peanuts, red wine	polyphenol	Jia et al. (2021)
1,2,3,4,6-penta-O-galloyl-β-D-glucopyranose	GAPDH	inhibit	colon cancer	natural plants	tannin family compound	Li et al. (2022b)

Some natural products trigger a selective yet potent host immune reaction against cancer cells, particularly given the interest in strategies which could improve response rates to immune checkpoint inhibitors by turning “cold” tumours “hot” (Galon and Bruni, 2019). Studies showed that glycolytic activity enhances PD-L1 expression on tumor cells and promotes anti-PD-1/PD-L1 immunotherapy response (Jiang et al., 2019). Natural products such as curcumin, the main active ingredient of turmeric, enhance the antitumor efficacy of sorafenib through activating immune function, downregulating EMT, suppressing anaerobic glycolysis and decreasing the lipid synthesis in IL-6/JAK/STAT3, IL-1β/NF-κB and PI3K/AKT pathway (Man et al., 2020). Co-delivery of PD-L1 siRNA and resveratrol, natural polyphenol detected in more than 70 plant species, especially in grapes’ skin and seeds, showed boost of anti-tumor immune response by modulation of TME *via* balancing glucose metabolic pathways of glycolysis and mitochondrial OXPHOS (Jia et al., 2021). 1,2,3,4,6-penta-O-galloyl-β-D-glucopyranose, a major component of the root of P. suffruiticosa inhibits LPS-stimulated macrophage activation through specific downregulation of GAPDH-dependent glucose consumption and lactate production (Li et al., 2022a).

### 2.2 The chemical classifications and effects of natural product targeting glycolysis

Natural products have received remarkable attention as anticancer agents that regulate glycolysis of cancer cells. Plant-derived bioactivecompounds-including polyphenols, alkaloids, quinones and terpenoids show promise as useful anticancer agents.

Polyphenols are a complex class of plant secondary metabolites consisting of an aromatic ring with at least one hydroxyl group, such as phenolic acids, cinnamic acids, coumarins, flavonoids, xanthones and stilbenes, comprised phenolic groups. Phenolic compounds, like quercetin (Jia et al., 2018), curcumin (Man et al., 2020), morusin (Cho et al., 2021), epigallocatechin gallate (Chen et al., 2022a), oleuropein (Ruzzolini et al., 2020), inhibit glucose and lactate cellular uptake, downregulate PKM2 and GLUT1 expression, and inhibit MCT1-mediated lactate reuptake in cancer treatments. Polyphenols, such resveratrol (Brockmueller et al., 2021b), salvianolic acid B (Wei et al., 2018a), and morusin (Cho et al., 2021), regulate glycolysis by down-regulating PI3K and AKT expression. Curcumin (Siddiqui et al., 2018a) decreases the Warburg effect through decreasing the expression of mTOR in cancer cells.

Alkaloids are a large and complex group of cyclic compounds that contain a nitrogen-bearing molecule. Dauricine, an isoquinoline alkaloid, downregulates the expression of HK2 and PKM2 in hepatocellular carcinoma cells (Li W. et al., 2018). Scopolin inhibits glycolysis by inhibiting PGK2, GPI and GPD2 in hepatocellular carcinoma (Wang et al., 2022). Berberine decelerates glucose metabolism *via* suppression of mTOR-dependent HIF-1α protein synthesis in colon cancer cells (Mao et al., 2018a).

Quinone is an aromatic compound with two carbonyl functional groups in the same six-membered ring. Emodin (Wang K. J. et al., 2021) down-regulates the expression of GLUT1. Tanshinone IIA (Li M. et al., 2020) and rhein (Wu et al., 2019) decrease HK2 expression. The important glycolytic enzyme PKM2 is inhibited by shikonin (Wang et al., 2018). Shikonin (Zhang et al., 2020) and emodin (Wang K. J. et al., 2021) also inhibit the PI3K/AKT signal pathway. Tanshinone IIA (Li M. et al., 2020) down-regulates AKT and the c-MYC oncogene expression.

Terpenoids, also known as isoprenoids or terpenes, usually have a cyclic structure. The triterpenoid molecules cucurbitacin D (Sikander et al., 2019) and *a*-Hederin (Fang et al., 2021a) down-regulate GLUT1 expression. HK2 was inhibited by tanshinone IIA (Li M. et al., 2020), triptolide (Hamdi et al., 2018) and oleanolic acid (Li et al., 2019). Triptolide (Hamdi et al., 2018) and tanshinone IIA (Li M. et al., 2020) both also decrease the expression of AKT. Moreover, the oncogene HIF-1α is inhibited by astragaloside IV (Zhang et al., 2018) and oleanolic acid (Li et al., 2019).

### 2.3 Natural products in clinical research

More than 100 plant and animal based natural compounds have been used in clinical treatment. From 1981 to 2019, about 40% of anticancer drugs were derived in part or whole from natural sources (Newman and Cragg, 2020), including (-)-β-elemene, paclitaxel, hydroxycamptothecin, camptothecins, colchicine and artemisinin. Morphine, the alkaloids mainly produced in the opium poppy (Papaver somniferum), marketed by Merck in 1826, is the first commercial pure natural product introduced for blocking moderate to severe pain that may be acute or chronic. Fermented wheat germ extract (FWGE), derived from the germ of the wheat plant, interferes with anaerobic glycolysis, the pentose cycle and ribonucleotide reductase and has significant antiproliferative effects, killings tumor cells by the induction of apoptosis *via* the caspase-poly [ADP-ribose] polymerase-pathway. It indicated a significant benefit for patients treated with the chemotherapy drug dacarbazine in combination with FWGE in terms of progression free survival and overall survival according to clinical data from a randomized phase II trial in melanoma patients (Mueller and Voigt, 2011). Silibinin is an extract from the medicinal plant Silybum marianum (milk thistle) reported to inhibit tumor aerobic glycolysis and alter PD-L1 expression by interfering with HIF-1α/LDH-A mediated cell metabolism in nasopharyngeal carcinoma (Sellam et al., 2020). Silibinin inhibits growth of prostate cancer cells by targeting the epidermal growth factor receptor (EGFR), insulin-like growth factor-1 receptor (IGF-1R) and NF-kappaB (nuclear factor-kappa B) pathways in prostate cancer. Clinical trials in human prostate cancer patients are ongoing (Singh and Agarwal, 2006). ACT001, derived from parthenolide, is an orphan drug currently in clinical trials for the treatment of glioblastoma (Zhang T. et al., 2022). It selectively activates PKM2 through covalent binding at residue cysteine 424 to promote tetramer formation and inhibit tumour metabolism (Li J. et al., 2018). Rhizoma Paridis Saponins (RPS), the major active component of Rhizoma Paridis, played an antitumor role in many clinical indications (Man et al., 2014) through regulation of glycolytic and lipid metabolism (Yao et al., 2018). Dauricine, the major bioactive component isolated from the roots of Menispermum dauricum D.C, has shown promising pharmacological activities with a great potential for clinical use, inhibiting glucose glycolysis and increasing oxidative phosphorylation by downregulating the expression of HK2 and PKM2 directly targeted by miR-199a in hepatocellular carcinoma cells (Li W. et al., 2018). Resveratrol, a polyphenol phytoalexin present in a variety of plant species, has been reported to have beneficial effects in tumor prevention, daily doses of resveratrol at 0.5 or 1.0 g produce levels in the human gastrointestinal tract that are of an order of magnitude sufficient to elicit anticarcinogenic effects (Patel et al., 2010). Curcumin, found primarily in turmeric, has characters of safety, tolerability, and non-toxicity at high doses. Circumin has exhibited activities against numerous cancer types in human clinical trials, including breast cancer, colorectal cancer, lung cancer, pancreatic cancer, prostate cancer, multiple myeloma, oral cancer and head and neck squamous cell carcinoma (Gupta et al., 2013). *ß*-Elemene is a bioactive triterpenoid derived from various plants. Qureshi et al. analyzed the results of clinical trials using *ß*-elemene to demonstrate that it regulates cancer progression and metastasis *via* various signal transduction cascades, including tumor necrosis factor related apoptosis-inducing ligand, signal transducers and activators of transcription, transforming growth factor/SMAD, NOTCH, and mammalian target of rapamycin pathways (Qureshi et al., 2019). Tanshinone IIA, isolated from the roots and rhizomes of the Chinese medicinal herb *Salvia miltiorrhiza* Bunge (Danshen), is currently used to treat patients with cardiovascular system abnormalities, diabetes, apoplexy, arthritis, sepsis and other diseases in China and neighboring countries. It has also been reported to have antitumor effects (Fang et al., 2020). Minnelide, a water-soluble prodrug of triptolide that is isolated from a Chinese medicinal herb, is currently in phase II clinical trials for treatment of pancreatic cancer. Only a few patients treated with Minnelide have been evaluated in phase I clinical trials. Plasma levels of the agent can be achieved, giving responses in patients with very refractory gastric or pancreatic cancer, and the agent can be given with a margin of safety (Noel et al., 2019). Russo et al. reported that sulforaphane modulates cellular homeostasis through the activation of the transcription factor Nrf2. Five of 20 completed or ongoing clinical trial from ClinicalTrials.gov were cancer related and partially confirm the promising anticancer potential of sulforaphane observed in pre-clinical experiments (Russo et al., 2018). Epigallocatechin-3-gallate, an active compound of green tea, modulates multiple molecular targets and inhibits the pathogenesis of cancer through inhibition of initiation, promotion and progression. Clinical trials on human subjects confirm that Epigallocatechin-3-gallate plays a role in various cancer prevention, including prostate cancer, urinary bladder cancer, head and neck cancer, breast cancer, ovarian cancer, and lung cancer (Almatroodi et al., 2020).

Natural products that target glycolysis to suppress tumor progression are used in the rarely currently and this is for a number of reasons. Firstly, many natural products are non-selective, and the translational potential of these glycolytic inhibitors is limited. Secondly, many immune cells such as T cells require high levels of glucose for their effector function, while drugs targeting tumor glycolysis will have undesirable effects on the glycolysis of tumor associated immune cells. Thirdly, low oral bioavailability limits the application of these products. Thus, as the value of these products is better appreciated, it will become necessary to invest in determining effective modifications that can overcome the drawbacks currently limiting their use. It is also vital to note, in the current climate, that bringing a natural product into clinical development requires a sustainable and economically viable supply of sufficient quantities of the natural product.

## 3 Conclusion and future perspectives

Natural product targeting glycolysis can effectively inhibit the development of tumors and provide an approach for the effective treatment of various cancers.

With respect to the tumor itself, tumor cells located in a nutrient-rich environment exhibit low or no sensitivity to targeted metabolic inhibitors (Ayuso et al., 2020). Tumors may judiciously use their environment to boost the body’s metabolism, providing the material basis for escape from the immune system (Kooshki et al., 2022). However, high glycolytic flux depends on glycolysis-related genes (Massari et al., 2016). Thus, targeting glycolysis is beneficial to the treatment of tumor.

Metabolic reprogramming is a central hallmark of cancer and is critical for tumorigenesis and tumor progression (Liu D. et al., 2021). Natural products can inhibit the proto-oncogene MYC, which ultimately suppresses HIF-1α-mediated metabolic reprogramming towards a glycolytic phenotype. Natural products have advantages in ameliorating cancer cell metabolic reprogramming by their poly-pharmacological actions. In the glycolysis signaling pathway, activation of AMPK and inhibition of PI3K/AKT/mTOR are the key targets of these products.

Compared to chemotherapy, natural products have the advantage of availability, high efficacy, and low toxicity. However, “natural” is not equivalent to “safe”, and the toxicity still severely hinders the wide use of natural products. Indeed, natural products possess a broad spectrum of chemical functional groups, some of which are recognized as structural alerts for toxicologically-based chronic effects, such as the chemical initiation of carcinogenesis. This demonstrates the importance of evaluating their potential toxicity. Metabolomics and *in silico* models have been used to evaluate for toxicity of natural products (Chen et al., 2016; Abdel-Wahab et al., 2021), but a more comprehensive understanding of their toxicity is urgently required.

This review has highlighted that natural products target glycolysis through regulating glycolytic enzymes and related proteins, oncogenes and glycolytic signaling pathways. More and more Chinese medicines have been proved to be widely used as adjuvant therapy after surgery, chemotherapy, radiotherapy or other types of treatment for cancer, alleviating various side effects caused by chemotherapy, such as gene mutation, cytotoxicity and drug resistance, showing promising therapeutic effects (Xiang et al., 2019) in clinical treatment. Continued exploration of the effective targets of natural products in glycolysis is required to obtain a more comprehensive picture of the mechanisms involved and the potential therapeutic targets.

## Data Availability

The original contributions presented in the study are included in the article/Supplementary Material, further inquiries can be directed to the corresponding authors.

## References

[B1] Abdel-Wahab A. A. Effat H. Mahrous E. A. Ali M. A. Al-Shafie T. A. (2021). A licorice roots extract induces apoptosis and cell cycle arrest and improves metabolism via regulating MiRNAs in liver cancer cells. Nutr. Cancer 73 (6), 1047–1058. 10.1080/01635581.2020.1783329 32578448

[B2] Afrin S. Giampieri F. Gasparrini M. Forbes-Hernández T. Y. Cianciosi D. Reboredo-Rodriguez P. (2018). The inhibitory effect of Manuka honey on human colon cancer HCT-116 and LoVo cell growth. Part 2: Induction of oxidative stress, alteration of mitochondrial respiration and glycolysis, and suppression of metastatic ability. Food Funct. 9 (4), 2158–2170. 10.1039/c8fo00165k 29644357

[B3] Akter R. Afrose A. Rahman M. R. Chowdhury R. Nirzhor S. S. R. Khan R. I. (2021). A comprehensive analysis into the therapeutic application of natural products as SIRT6 modulators in alzheimer's disease, aging, cancer, inflammation, and diabetes. Int. J. Mol. Sci. 22 (8), 4180. 10.3390/ijms22084180 33920726 PMC8073883

[B4] Almatroodi S. A. Almatroudi A. Khan A. A. Alhumaydhi F. A. Alsahli M. A. Rahmani A. H. (2020). Potential therapeutic targets of epigallocatechin gallate (EGCG), the most abundant catechin in green tea, and its role in the therapy of various types of cancer. Molecules 25 (14), E3146. 10.3390/molecules25143146 32660101 PMC7397003

[B5] Atanasov A. G. Waltenberger B. Pferschy-Wenzig E. M. Linder T. Wawrosch C. Uhrin P. (2015). Discovery and resupply of pharmacologically active plant-derived natural products: A review. Biotechnol. Adv. 33 (8), 1582–1614. 10.1016/j.biotechadv.2015.08.001 26281720 PMC4748402

[B6] Atanasov A. G. Zotchev S. B. Dirsch V. M. Supuran C. T. (2021). Natural products in drug discovery: Advances and opportunities. Nat. Rev. Drug Discov. 20 (3), 200–216. 10.1038/s41573-020-00114-z 33510482 PMC7841765

[B7] Ayuso J. M. Rehman S. Farooqui M. Virumbrales-Muñoz M. Setaluri V. Skala M. C. (2020). Microfluidic tumor-on-a-chip model to study tumor metabolic vulnerability. Int. J. Mol. Sci. 21 (23), E9075. 10.3390/ijms21239075 33260673 PMC7730115

[B8] Beltinger C. (2019). LDHA and LDHB are dispensable for aerobic glycolysis in neuroblastoma cells while promoting their aggressiveness. J. Biol. Chem. 294 (1), 66. 10.1074/jbc.L118.006717 30610122 PMC6322903

[B9] Benito-Cuesta I. Ordonez-Gutierrez L. Wandosell F. (2021). AMPK activation does not enhance autophagy in neurons in contrast to MTORC1 inhibition: Different impact on beta-amyloid clearance. Autophagy 17 (3), 656–671. 10.1080/15548627.2020.1728095 32075509 PMC8032230

[B10] Bonuccelli G. Sotgia F. Lisanti M. P. (2018). Matcha green tea (MGT) inhibits the propagation of cancer stem cells (CSCs), by targeting mitochondrial metabolism, glycolysis and multiple cell signalling pathways. Aging (Albany NY) 10 (8), 1867–1883. 10.18632/aging.101483 30153655 PMC6128439

[B11] Brockmueller A. Sameri S. Liskova A. Zhai K. Varghese E. Samuel S. M. (2021a). Resveratrol's anti-cancer effects through the modulation of tumor glucose metabolism. Cancers (Basel) 13 (2), E188. 10.3390/cancers13020188 33430318 PMC7825813

[B12] Brockmueller A. Sameri S. Liskova A. Zhai K. Varghese E. Samuel S. M. (2021b). Resveratrol's anti-cancer effects through the modulation of tumor glucose metabolism. Cancers (Basel) 13 (2), E188. 10.3390/cancers13020188 33430318 PMC7825813

[B13] Cai T. Zhang C. Zeng X. Zhao Z. Yan Y. Yu X. (2019). Protective effects of Weipixiao decoction against MNNG-induced gastric precancerous lesions in rats. Biomed. Pharmacother. 120, 109427. 10.1016/j.biopha.2019.109427 31648165

[B14] Cao H. Wang D. Sun P. Chen L. Feng Y. Gao R. (2021). Zhoushi Qi Ling decoction represses docetaxel resistance and glycolysis of castration-resistant prostate cancer via regulation of SNHG10/miR-1271-5p/TRIM66 axis. Aging (Albany NY) 13 (19), 23096–23107. 10.18632/aging.203602 34613933 PMC8544336

[B15] Carrasco-Pozo C. Tan K. N. Rodriguez T. Avery V. M. (2019). The molecular effects of sulforaphane and capsaicin on metabolism upon androgen and Tip60 activation of androgen receptor. Int. J. Mol. Sci. 20 (21), E5384. 10.3390/ijms20215384 31671779 PMC6861939

[B16] Chen D. Q. Chen H. Chen L. Tang D. D. Miao H. Zhao Y. Y. (2016). Metabolomic application in toxicity evaluation and toxicological biomarker identification of natural product. Chem. Biol. Interact. 252, 114–130. 10.1016/j.cbi.2016.03.028 27041073

[B17] Chen R. L. Wang Z. Huang P. Sun C. H. Yu W. Y. Zhang H. H. (2021a). Isovitexin potentiated the antitumor activity of cisplatin by inhibiting the glucose metabolism of lung cancer cells and reduced cisplatin-induced immunotoxicity in mice. Int. Immunopharmacol. 94, 107357. 10.1016/j.intimp.2020.107357 33715980

[B18] Chen S. Nishi M. Morine Y. Shimada M. Tokunaga T. Kashihara H. (2022a). Epigallocatechin3gallate hinders metabolic coupling to suppress colorectal cancer malignancy through targeting aerobic glycolysis in cancerassociated fibroblasts. Int. J. Oncol. 60 (2), 19. 10.3892/ijo.2022.5309 35029285 PMC8776327

[B19] Chen Y. Zhang J. Zhang M. Song Y. Zhang Y. Fan S. (2021b). Baicalein resensitizes tamoxifen-resistant breast cancer cells by reducing aerobic glycolysis and reversing mitochondrial dysfunction via inhibition of hypoxia-inducible factor-1α. Clin. Transl. Med. 11 (11), e577. 10.1002/ctm2.577 34841716 PMC8567056

[B20] Cho A. R. Park W. Y. Lee H. J. Sim D. Y. Im E. Park J. E. (2021). Antitumor effect of morusin via G1 arrest and antiglycolysis by AMPK activation in hepatocellular cancer. Int. J. Mol. Sci. 22 (19), 10619. 10.3390/ijms221910619 34638959 PMC8508967

[B21] Daddiouaissa D. Amid A. Abdullah Sani M. S. Elnour A. A. M. (2021). Evaluation of metabolomics behavior of human colon cancer HT29 cell lines treated with ionic liquid graviola fruit pulp extract. J. Ethnopharmacol. 270, 113813. 10.1016/j.jep.2021.113813 33444719

[B22] Dang C. V. Le A. Gao P. (2009). MYC-induced cancer cell energy metabolism and therapeutic opportunities. Clin. Cancer Res. 15 (21), 6479–6483. 10.1158/1078-0432.CCR-09-0889 19861459 PMC2783410

[B23] Ding Y. Xue Q. Liu S. Hu K. Wang D. Wang T. (2020). Identification of parthenolide dimers as activators of pyruvate kinase M2 in xenografts of glioblastoma multiforme *in vivo* . J. Med. Chem. 63 (4), 1597–1611. 10.1021/acs.jmedchem.9b01328 31977207

[B24] Fan H. Y. Zhu Z. L. Xian H. C. Wang H. F. Chen B. J. Tang Y. J. (2021). Insight into the molecular mechanism of podophyllotoxin derivatives as anticancer drugs. Front. Cell Dev. Biol. 9, 709075. 10.3389/fcell.2021.709075 34447752 PMC8383743

[B25] Fang C. Liu Y. Chen L. Luo Y. Cui Y. Zhang N. (2021a). α-Hederin inhibits the growth of lung cancer A549 cells *in vitro* and *in vivo* by decreasing SIRT6 dependent glycolysis. Pharm. Biol. 59 (1), 11–20. 10.1080/13880209.2020.1862250 33356727 PMC7782159

[B26] Fang Z. Y. Zhang M. Liu J. N. Zhao X. Zhang Y. Q. Fang L. (2020). Tanshinone IIA: A review of its anticancer effects. Front. Pharmacol. 11, 611087. 10.3389/fphar.2020.611087 33597880 PMC7883641

[B27] Feng J. Wu L. Ji J. Chen K. Yu Q. Zhang J. (2019). PKM2 is the target of proanthocyanidin B2 during the inhibition of hepatocellular carcinoma. J. Exp. Clin. Cancer Res. 38 (1), 204. 10.1186/s13046-019-1194-z 31101057 PMC6525465

[B28] Galon J. Bruni D. (2019). Approaches to treat immune hot, altered and cold tumours with combination immunotherapies. Nat. Rev. Drug Discov. 18 (3), 197–218. 10.1038/s41573-018-0007-y 30610226

[B29] Gao J. L. Chen Y. G. (2015). Natural compounds regulate glycolysis in hypoxic tumor microenvironment. Biomed. Res. Int. 2015, 354143. 10.1155/2015/354143 25685782 PMC4317583

[B30] Gao Y. Zhou H. Liu G. Wu J. Yuan Y. Shang A. (2022). Tumor microenvironment: Lactic acid promotes tumor development. J. Immunol. Res. 2022, 3119375. 10.1155/2022/3119375 35733921 PMC9207018

[B31] Guo H. Wan B. Wang J. Zhang J. Yao W. Shen Z. (2019). Astragalus saponins inhibit cell growth, aerobic glycolysis and attenuate the inflammatory response in a DSS-induced colitis model. Int. J. Mol. Med. 43 (2), 1041–1048. 10.3892/ijmm.2018.4036 30569176

[B32] Gupta S. C. Patchva S. Aggarwal B. B. (2013). Therapeutic roles of curcumin: Lessons learned from clinical trials. AAPS J. 15 (1), 195–218. 10.1208/s12248-012-9432-8 23143785 PMC3535097

[B33] Hamdi A. M. Jiang Z. Z. Guerram M. Yousef B. A. Hassan H. M. Ling J. W. (2018). Biochemical and computational evaluation of Triptolide-induced cytotoxicity against NSCLC. Biomed. Pharmacother. 103, 1557–1566. 10.1016/j.biopha.2018.04.198 29864943

[B34] Han J. H. Kim M. Kim H. J. Jang S. B. Bae S. J. Lee I. K. (2021). Targeting lactate dehydrogenase A with catechin resensitizes SNU620/5FU gastric cancer cells to 5-fluorouracil. Int. J. Mol. Sci. 22 (10), 5406. 10.3390/ijms22105406 34065602 PMC8161398

[B35] He P. Xu Y. Wang Z. (2020). LncRNA SNHG10 increases the methylation of miR-218 gene to promote glucose uptake and cell proliferation in osteosarcoma. J. Orthop. Surg. Res. 15 (1), 353. 10.1186/s13018-020-01865-6 32843060 PMC7448318

[B36] Herzig S. Shaw R. J. (2018). Ampk: Guardian of metabolism and mitochondrial homeostasis. Nat. Rev. Mol. Cell Biol. 19 (2), 121–135. 10.1038/nrm.2017.95 28974774 PMC5780224

[B37] Holman G. D. (2020). Structure, function and regulation of mammalian glucose transporters of the SLC2 family. Pflugers Arch. 472 (9), 1155–1175. 10.1007/s00424-020-02411-3 32591905 PMC7462842

[B38] Hong J. Xie Z. Yang F. Jiang L. Jian T. Wang S. (2022). Erianin suppresses proliferation and migration of cancer cells in a pyruvate carboxylase-dependent manner. Fitoterapia 157, 105136. 10.1016/j.fitote.2022.105136 35093481

[B39] Hua H. Kong Q. Zhang H. Wang J. Luo T. Jiang Y. (2019). Targeting mTOR for cancer therapy. J. Hematol. Oncol. 12 (1), 71. 10.1186/s13045-019-0754-1 31277692 PMC6612215

[B40] Huang S. C. Smith A. M. Everts B. Colonna M. Pearce E. L. Schilling J. D. (2016). Metabolic reprogramming mediated by the mTORC2-IRF4 signaling Axis is essential for macrophage alternative activation. Immunity 45 (4), 817–830. 10.1016/j.immuni.2016.09.016 27760338 PMC5535820

[B41] Ji H. F. Li X. J. Zhang H. Y. (2009). Natural products and drug discovery. Can thousands of years of ancient medical knowledge lead us to new and powerful drug combinations in the fight against cancer and dementia? EMBO Rep. 10 (3), 194–200. 10.1038/embor.2009.12 19229284 PMC2658564

[B42] Jia L. Gao Y. Zhou T. Zhao X. L. Hu H. Y. Chen D. W. (2021). Enhanced response to PD-L1 silencing by modulation of TME via balancing glucose metabolism and robust co-delivery of siRNA/Resveratrol with dual-responsive polyplexes. Biomaterials 271, 120711. 10.1016/j.biomaterials.2021.120711 33592352

[B43] Jia L. Huang S. Yin X. Zan Y. Guo Y. Han L. (2018). Quercetin suppresses the mobility of breast cancer by suppressing glycolysis through Akt-mTOR pathway mediated autophagy induction. Life Sci. 208, 123–130. 10.1016/j.lfs.2018.07.027 30025823

[B44] Jiang T. Huang J. B. Xu C. Y. Lv Y. L. Lu J. Zhao Z. Q. (2022). Arsenic trioxide cooperate cryptotanshinone exerts antitumor effect by medicating macrophage polarization through glycolysis. J. Immunol. Res. 2022, 2619781. 10.1155/2022/2619781 35178457 PMC8846972

[B45] Jiang Z. Liu Z. Li M. Chen C. Wang X. (2019). Increased glycolysis correlates with elevated immune activity in tumor immune microenvironment. EBioMedicine 42, 431–442. 10.1016/j.ebiom.2019.03.068 30935888 PMC6491961

[B46] Jiao L. Wang S. Zheng Y. Wang N. Yang B. Wang D. (2019a). Betulinic acid suppresses breast cancer aerobic glycolysis via caveolin-1/NF-κB/c-Myc pathway. Biochem. Pharmacol. 161, 149–162. 10.1016/j.bcp.2019.01.016 30684465

[B47] Jin H. Dai J. Chen X. Liu J. Zhong D. Gu Y. (2010). Pulmonary toxicity and metabolic activation of dauricine in CD-1 mice. J. Pharmacol. Exp. Ther. 332 (3), 738–746. 10.1124/jpet.109.162297 20008063

[B48] Jin J. Qiu S. Wang P. Liang X. Huang F. Wu H. (2019a). Cardamonin inhibits breast cancer growth by repressing HIF-1α-dependent metabolic reprogramming. J. Exp. Clin. Cancer Res. 38 (1), 377. 10.1186/s13046-019-1351-4 31455352 PMC6712736

[B49] Jin S. Chang X. C. Wen J. Yang J. Ao N. Zhang K. Y. (2021). Decarboxylated osteocalcin, a possible drug for type 2 diabetes, triggers glucose uptake in MG63 cells. World J. Diabetes 12 (7), 1102–1115. 10.4239/wjd.v12.i7.1102 34326958 PMC8311485

[B50] Kalezic A. Udicki M. Srdic Galic B. Aleksic M. Korac A. Jankovic A. (2021). Tissue-specific Warburg effect in breast cancer and cancer-associated adipose tissue-relationship between AMPK and glycolysis. Cancers (Basel) 13 (11), 2731. 10.3390/cancers13112731 34073074 PMC8198826

[B51] Kim S. L. Park Y. R. Lee S. T. Kim S. W. (2017). Parthenolide suppresses hypoxia-inducible factor-1α signaling and hypoxia induced epithelial-mesenchymal transition in colorectal cancer. Int. J. Oncol. 51 (6), 1809–1820. 10.3892/ijo.2017.4166 29075793

[B52] Kooshki L. Mahdavi P. Fakhri S. Akkol E. K. Khan H. (2022). Targeting lactate metabolism and glycolytic pathways in the tumor microenvironment by natural products: A promising strategy in combating cancer. Biofactors 48 (2), 359–383. 10.1002/biof.1799 34724274

[B53] Koppenol W. H. Bounds P. L. Dang C. V. (2011). Otto Warburg's contributions to current concepts of cancer metabolism. Nat. Rev. Cancer 11 (5), 325–337. 10.1038/nrc3038 21508971

[B54] Korga A. Ostrowska M. Jozefczyk A. Iwan M. Wojcik R. Zgorka G. (2019). Apigenin and hesperidin augment the toxic effect of doxorubicin against HepG2 cells. BMC Pharmacol. Toxicol. 20 (1), 22. 10.1186/s40360-019-0301-2 31053173 PMC6499973

[B55] Kottakis F. Bardeesy N. (2012). LKB1-AMPK axis revisited. Cell Res. 22 (12), 1617–1620. 10.1038/cr.2012.108 22801477 PMC3515750

[B56] Lee P. Chandel N. S. Simon M. C. (2020). Cellular adaptation to hypoxia through hypoxia inducible factors and beyond. Nat. Rev. Mol. Cell Biol. 21 (5), 268–283. 10.1038/s41580-020-0227-y 32144406 PMC7222024

[B57] Li H. Hu S. Pang Y. Li M. Chen L. Liu F. (2018a). Bufalin inhibits glycolysis-induced cell growth and proliferation through the suppression of Integrin β2/FAK signaling pathway in ovarian cancer. Am. J. Cancer Res. 8 (7), 1288–1296. 30094101 PMC6079152

[B58] Li J. Li S. Guo J. Li Q. Long J. Ma C. (2018c). Natural product micheliolide (MCL) irreversibly activates pyruvate kinase M2 and suppresses leukemia. J. Med. Chem. 61 (9), 4155–4164. 10.1021/acs.jmedchem.8b00241 29641204 PMC5949721

[B59] Li J. Zou Y. Pei M. Zhang Y. Jiang Y. (2021). Berberine inhibits the Warburg effect through TET3/miR-145/HK2 pathways in ovarian cancer cells. J. Cancer 12 (1), 207–216. 10.7150/jca.48896 33391417 PMC7738813

[B60] Li M. Gao F. Zhao Q. Zuo H. Liu W. Li W. (2020a). Tanshinone IIA inhibits oral squamous cell carcinoma via reducing Akt-c-Myc signaling-mediated aerobic glycolysis. Cell Death Dis. 11 (5), 381. 10.1038/s41419-020-2579-9 32424132 PMC7235009

[B61] Li S. Li J. Dai W. Zhang Q. Feng J. Wu L. (2017). Genistein suppresses aerobic glycolysis and induces hepatocellular carcinoma cell death. Br. J. Cancer 117 (10), 1518–1528. 10.1038/bjc.2017.323 28926527 PMC5680469

[B62] Li W. Liao L. P. Song N. Liu Y. J. Ding Y. L. Zhang Y. Y. (2022a). Natural product 1, 2, 3, 4, 6-penta-O-galloyl-beta-D-glucopyranose is a reversible inhibitor of glyceraldehyde 3-phosphate dehydrogenase. Acta Pharmacol. Sin. 43 (2), 470–482. 10.1038/s41401-021-00653-0 33850276 PMC8792024

[B63] Li W. Liao L. P. Song N. Liu Y. J. Ding Y. L. Zhang Y. Y. (2022b). Natural product 1, 2, 3, 4, 6-penta-O-galloyl-β-D-glucopyranose is a reversible inhibitor of glyceraldehyde 3-phosphate dehydrogenase. Acta Pharmacol. Sin. 43 (2), 470–482. 10.1038/s41401-021-00653-0 33850276 PMC8792024

[B64] Li W. Qiu Y. Hao J. Zhao C. Deng X. Shu G. (2018d). Dauricine upregulates the chemosensitivity of hepatocellular carcinoma cells: Role of repressing glycolysis via miR-199a:HK2/PKM2 modulation. Food Chem. Toxicol. 121, 156–165. 10.1016/j.fct.2018.08.030 30171973

[B65] Li X. Sun J. Xu Q. Duan W. Yang L. Wu X. (2020b). Oxymatrine inhibits colorectal cancer metastasis via attenuating PKM2-mediated aerobic glycolysis. Cancer Manag. Res. 12, 9503–9513. 10.2147/cmar.S267686 33061637 PMC7534866

[B66] Li Y. Xu Q. Yang W. Wu T. Lu X. (2019). Oleanolic acid reduces aerobic glycolysis-associated proliferation by inhibiting yes-associated protein in gastric cancer cells. Gene 712, 143956. 10.1016/j.gene.2019.143956 31271843

[B67] Liu D. Jin X. Yu G. Wang M. Liu L. Zhang W. (2021a). Oleanolic acid blocks the purine salvage pathway for cancer therapy by inactivating SOD1 and stimulating lysosomal proteolysis. Mol. Ther. Oncolytics 23, 107–123. 10.1016/j.omto.2021.08.013 34703880 PMC8505360

[B68] Liu W. Zhao Z. M. Liu Y. L. Pan H. F. Lin L. Z. (2019). Weipiling ameliorates gastric precancerous lesions in Atp4a(-/-) mice. BMC Complement. Altern. Med. 19 (1), 318. 10.1186/s12906-019-2718-y 31744486 PMC6862855

[B69] Liu X. Wang C. Li S. Qu L. Yin F. Lu D. (2021b). Parthenolide derivatives as PKM2 activators showing potential in colorectal cancer. J. Med. Chem. 64 (23), 17304–17325. 10.1021/acs.jmedchem.1c01380 34847663

[B70] Lu Q. Y. Zhang L. Yee J. K. Go V. W. Lee W. N. (2015). Metabolic consequences of LDHA inhibition by epigallocatechin gallate and oxamate in MIA PaCa-2 pancreatic cancer cells. Metabolomics 11 (1), 71–80. 10.1007/s11306-014-0672-8 26246802 PMC4523095

[B71] Ma S. Wang F. Zhang C. Wang X. Wang X. Yu Z. (2020). Cell metabolomics to study the function mechanism of Cyperus rotundus L. on triple-negative breast cancer cells. BMC Complement. Med. Ther. 20 (1), 262. 10.1186/s12906-020-02981-w 32843016 PMC7449030

[B72] Man S. Fan W. Liu Z. Gao W. Li Y. Zhang L. (2014). Antitumor pathway of Rhizoma Paridis Saponins based on the metabolic regulatory network alterations in H22 hepatocarcinoma mice. Steroids 84, 17–21. 10.1016/j.steroids.2014.03.005 24642033

[B73] Man S. Yao J. Lv P. Liu Y. Yang L. Ma L. (2020). Curcumin-enhanced antitumor effects of sorafenib via regulating the metabolism and tumor microenvironment. Food Funct. 11 (7), 6422–6432. 10.1039/c9fo01901d 32613952

[B74] Manning B. D. Toker A. (2017). AKT/PKB signaling: Navigating the network. Cell 169 (3), 381–405. 10.1016/j.cell.2017.04.001 28431241 PMC5546324

[B75] Mao L. Chen Q. Gong K. Xu X. Xie Y. Zhang W. (2018a). Berberine decelerates glucose metabolism via suppression of mTOR-dependent HIF-1α protein synthesis in colon cancer cells. Oncol. Rep. 39 (5), 2436–2442. 10.3892/or.2018.6318 29565467

[B76] Massari F. Ciccarese C. Santoni M. Iacovelli R. Mazzucchelli R. Piva F. (2016). Metabolic phenotype of bladder cancer. Cancer Treat. Rev. 45, 46–57. 10.1016/j.ctrv.2016.03.005 26975021

[B77] Miao G. Han J. Zhang J. Wu Y. Tong G. (2019). Targeting pyruvate kinase M2 and hexokinase II, pachymic acid impairs glucose metabolism and induces mitochondrial apoptosis. Biol. Pharm. Bull. 42 (1), 123–129. 10.1248/bpb.b18-00730 30381614

[B78] Mueller T. Voigt W. (2011). Fermented wheat germ extract--nutritional supplement or anticancer drug? Nutr. J. 10, 89. 10.1186/1475-2891-10-89 21892933 PMC3179707

[B79] Mukhopadhyay S. Vander Heiden M. G. McCormick F. (2021). The metabolic landscape of RAS-driven cancers from biology to therapy. Nat. Cancer 2 (3), 271–283. 10.1038/s43018-021-00184-x 33870211 PMC8045781

[B80] Nakayama J. Konno Y. Maruyama A. Tomita M. Makinoshima H. (2022). Cinnamon bark extract suppresses metastatic dissemination of cancer cells through inhibition of glycolytic metabolism. J. Nat. Med. 76 (3), 686–692. 10.1007/s11418-022-01624-3 35445961

[B81] Nalini D. Selvaraj J. Kumar G. S. (2020). Herbal nutraceuticals: Safe and potent therapeutics to battle tumor hypoxia. J. Cancer Res. Clin. Oncol. 146 (1), 1–18. 10.1007/s00432-019-03068-x 31724069 PMC11804421

[B82] Newman D. J. Cragg G. M. (2012). Natural products as sources of new drugs over the 30 years from 1981 to 2010. J. Nat. Prod. 75 (3), 311–335. 10.1021/np200906s 22316239 PMC3721181

[B83] Newman D. J. Cragg G. M. (2007). Natural products as sources of new drugs over the last 25 years. J. Nat. Prod. 70 (3), 461–477. 10.1021/np068054v 17309302

[B84] Newman D. J. Cragg G. M. (2020). Natural products as sources of new drugs over the nearly four decades from 01/1981 to 09/2019. J. Nat. Prod. 83 (3), 770–803. 10.1021/acs.jnatprod.9b01285 32162523

[B85] Noel P. Von Hoff D. D. Saluja A. K. Velagapudi M. Borazanci E. Han H. (2019). Triptolide and its derivatives as cancer therapies. Trends Pharmacol. Sci. 40 (5), 327–341. 10.1016/j.tips.2019.03.002 30975442

[B86] Obaidi I. Blanco Fernandez A. McMorrow T. (2022). Curcumin sensitises cancerous kidney cells to TRAIL induced apoptosis via let-7C mediated deregulation of cell cycle proteins and cellular metabolism. Int. J. Mol. Sci. 23 (17), 9569. 10.3390/ijms23179569 36076967 PMC9455736

[B87] Pan Y. Wang W. Huang S. Ni W. Wei Z. Cao Y. (2019a). Beta-elemene inhibits breast cancer metastasis through blocking pyruvate kinase M2 dimerization and nuclear translocation. J. Cell. Mol. Med. 23 (10), 6846–6858. 10.1111/jcmm.14568 31343107 PMC6787513

[B88] Pan Y. Zheng Q. Ni W. Wei Z. Yu S. Jia Q. (2019b). Breaking glucose transporter 1/pyruvate kinase M2 glycolytic loop is required for cantharidin inhibition of metastasis in highly metastatic breast cancer. Front. Pharmacol. 10, 590. 10.3389/fphar.2019.00590 31178738 PMC6544055

[B89] Park M. K. Ji J. Haam K. Han T. H. Lim S. Kang M. J. (2021b). Licochalcone A inhibits hypoxia-inducible factor-1α accumulation by suppressing mitochondrial respiration in hypoxic cancer cells. Biomed. Pharmacother. 133, 111082. 10.1016/j.biopha.2020.111082 33378978

[B90] Park M. K. Ji J. Haam K. Han T. H. Lim S. Kang M. J. (2021a). Licochalcone A inhibits hypoxia-inducible factor-1α accumulation by suppressing mitochondrial respiration in hypoxic cancer cells. Biomed. Pharmacother. 133, 111082. 10.1016/j.biopha.2020.111082 33378978

[B91] Patel K. R. Brown V. A. Jones D. J. Britton R. G. Hemingway D. Miller A. S. (2010). Clinical pharmacology of resveratrol and its metabolites in colorectal cancer patients. Cancer Res. 70 (19), 7392–7399. 10.1158/0008-5472.CAN-10-2027 20841478 PMC2948608

[B92] Qureshi M. Z. Attar R. Romero M. A. Sabitaliyevich U. Y. Nurmurzayevich S. B. Ozturk O. (2019). Regulation of signaling pathways by beta-elemene in cancer progression and metastasis. J. Cell. Biochem. 120 (8), 12091–12100. 10.1002/jcb.28624 30912190

[B93] Rani S. Roy S. Singh M. Kaithwas G. (2022). Regulation of transactivation at C-tad domain of HIF-1α by factor-inhibiting HIF-1α (FIH-1): A potential target for therapeutic intervention in cancer. Oxid. Med. Cell. Longev. 2022, 2407223. 10.1155/2022/2407223 35592530 PMC9113874

[B94] Russo M. Spagnuolo C. Russo G. L. Skalicka-Wozniak K. Daglia M. Sobarzo-Sanchez E. (2018). Nrf2 targeting by sulforaphane: A potential therapy for cancer treatment. Crit. Rev. Food Sci. Nutr. 58 (8), 1391–1405. 10.1080/10408398.2016.1259983 28001083

[B95] Ruzzolini J. Peppicelli S. Bianchini F. Andreucci E. Urciuoli S. Romani A. (2020). Cancer glycolytic dependence as a new target of olive leaf extract. Cancers (Basel) 12 (2), E317. 10.3390/cancers12020317 32013090 PMC7072393

[B96] Saunier E. Antonio S. Regazzetti A. Auzeil N. Laprevote O. Shay J. W. (2017). Resveratrol reverses the Warburg effect by targeting the pyruvate dehydrogenase complex in colon cancer cells. Sci. Rep. 7 (1), 6945. 10.1038/s41598-017-07006-0 28761044 PMC5537345

[B97] Sellam L. S. Zappasodi R. Chettibi F. Djennaoui D. Yahi-Ait Mesbah N. Amir-Tidadini Z. C. (2020). Silibinin down-regulates PD-L1 expression in nasopharyngeal carcinoma by interfering with tumor cell glycolytic metabolism. Arch. Biochem. Biophys. 690, 108479. 10.1016/j.abb.2020.108479 32679194 PMC8507490

[B98] Shankar Babu M. Mahanta S. Lakhter A. J. Hato T. Paul S. Naidu S. R. (2018). Lapachol inhibits glycolysis in cancer cells by targeting pyruvate kinase M2. PLoS One 13 (2), e0191419. 10.1371/journal.pone.0191419 29394289 PMC5796696

[B99] Shin N. Lee H. J. Sim D. Y. Im E. Park J. E. Park W. Y. (2021). Apoptotic effect of compound K in hepatocellular carcinoma cells via inhibition of glycolysis and Akt/mTOR/c-Myc signaling. Phytother. Res. 35 (7), 3812–3820. 10.1002/ptr.7087 33856720

[B100] Siddiqui F. A. Prakasam G. Chattopadhyay S. Rehman A. U. Padder R. A. Ansari M. A. (2018a). Curcumin decreases Warburg effect in cancer cells by down-regulating pyruvate kinase M2 via mTOR-HIF1α inhibition. Sci. Rep. 8 (1), 8323. 10.1038/s41598-018-25524-3 29844464 PMC5974195

[B101] Sikander M. Malik S. Chauhan N. Khan P. Kumari S. Kashyap V. K. (2019). Cucurbitacin D reprograms glucose metabolic network in prostate cancer. Cancers (Basel) 11 (3), E364. 10.3390/cancers11030364 30875788 PMC6469021

[B102] Singh K. B. Hahm E. R. Rigatti L. H. Normolle D. P. Yuan J. M. Singh S. V. (2018). Inhibition of glycolysis in prostate cancer chemoprevention by phenethyl isothiocyanate. Cancer Prev. Res. 11 (6), 337–346. 10.1158/1940-6207.Capr-17-0389 PMC5984685 29545400

[B103] Singh R. P. Agarwal R. (2006). Prostate cancer chemoprevention by silibinin: Bench to bedside. Mol. Carcinog. 45 (6), 436–442. 10.1002/mc.20223 16637061

[B104] Song W. Li D. Tao L. Luo Q. Chen L. (2020). Solute carrier transporters: The metabolic gatekeepers of immune cells. Acta Pharm. Sin. B 10 (1), 61–78. 10.1016/j.apsb.2019.12.006 31993307 PMC6977534

[B105] Sripriya N. Ranjith Kumar M. Udaya Prakash N. K. (2021). *In silico* evaluation of multispecies toxicity of natural compounds. Drug Chem. Toxicol. 44 (5), 480–486. 10.1080/01480545.2019.1614023 31111731

[B106] Steinberg G. R. Carling D. (2019). AMP-Activated protein kinase: The current landscape for drug development. Nat. Rev. Drug Discov. 18 (7), 527–551. 10.1038/s41573-019-0019-2 30867601

[B107] Sun L. T. Zhang L. Y. Shan F. Y. Shen M. H. Ruan S. M. (2021b). Jiedu Sangen decoction inhibits chemoresistance to 5-fluorouracil of colorectal cancer cells by suppressing glycolysis via PI3K/AKT/HIF-1α signaling pathway. Chin. J. Nat. Med. 19 (2), 143–152. 10.1016/s1875-5364(21)60015-8 33641785

[B108] Sun L. T. Zhang L. Y. Shan F. Y. Shen M. H. Ruan S. M. (2021a). Jiedu Sangen decoction inhibits chemoresistance to 5-fluorouracil of colorectal cancer cells by suppressing glycolysis via PI3K/AKT/HIF-1α signaling pathway. Chin. J. Nat. Med. 19 (2), 143–152. 10.1016/S1875-5364(21)60015-8 33641785

[B109] Sun Q. Yuan M. Wang H. Zhang X. Zhang R. Wang H. (2021c). PKM2 is the target of a multi-herb-combined decoction during the inhibition of gastric cancer progression. Front. Oncol. 11, 767116. 10.3389/fonc.2021.767116 34926270 PMC8675178

[B110] Sun Y. Chen Y. Xu M. Liu C. Shang H. Wang C. (2020). Shenmai injection supresses glycolysis and enhances cisplatin cytotoxicity in cisplatin-resistant A549/DDP cells via the AKT-mTOR-c-myc signaling pathway. Biomed. Res. Int. 2020, 9243681. 10.1155/2020/9243681 32685545 PMC7327568

[B111] Sur S. Nakanishi H. Flaveny C. Ippolito J. E. McHowat J. Ford D. A. (2019). Inhibition of the key metabolic pathways, glycolysis and lipogenesis, of oral cancer by bitter melon extract. Cell Commun. Signal. 17 (1), 131. 10.1186/s12964-019-0447-y 31638999 PMC6802351

[B112] Tailor D. Going C. C. Resendez A. Kumar V. Nambiar D. K. Li Y. (2021). Novel Aza-podophyllotoxin derivative induces oxidative phosphorylation and cell death via AMPK activation in triple-negative breast cancer. Br. J. Cancer 124 (3), 604–615. 10.1038/s41416-020-01137-4 33139797 PMC7851402

[B113] Tang W. Liu Z. L. Mai X. Y. Qi X. Li D. H. Gu Q. Q. (2020). Identification of Gliotoxin isolated from marine fungus as a new pyruvate kinase M2 inhibitor. Biochem. Biophys. Res. Commun. 528 (3), 594–600. 10.1016/j.bbrc.2020.05.139 32507600

[B114] Taş İ. Varlı M. Son Y. Han J. Kwak D. Yang Y. (2021). Physciosporin suppresses mitochondrial respiration, aerobic glycolysis, and tumorigenesis in breast cancer. Phytomedicine. 91, 153674. 10.1016/j.phymed.2021.153674 34333327

[B115] Trefts E. Shaw R. J. (2021). Ampk: Restoring metabolic homeostasis over space and time. Mol. Cell 81 (18), 3677–3690. 10.1016/j.molcel.2021.08.015 34547233 PMC8549486

[B116] Wang C. Zhou Q. Wu S. T. (2022). Scopolin obtained from Smilax China L. against hepatocellular carcinoma by inhibiting glycolysis: A network pharmacology and experimental study. J. Ethnopharmacol. 296, 115469. 10.1016/j.jep.2022.115469 35718053

[B117] Wang K. Huang W. Sang X. Wu X. Shan Q. Tang D. (2020). Atractylenolide I inhibits colorectal cancer cell proliferation by affecting metabolism and stemness via AKT/mTOR signaling. Phytomedicine 68, 153191. 10.1016/j.phymed.2020.153191 32135457

[B118] Wang K. J. Meng X. Y. Chen J. F. Wang K. Y. Zhou C. Yu R. (2021a). Emodin induced necroptosis and inhibited glycolysis in the renal cancer cells by enhancing ROS. Oxid. Med. Cell. Longev. 2021, 8840590. 10.1155/2021/8840590 33532038 PMC7837784

[B119] Wang K. X. Du G. H. Qin X. M. Gao L. (2021b). Compound Kushen Injection intervenes metabolic reprogramming and epithelial-mesenchymal transition of HCC via regulating β-catenin/c-Myc signaling. Phytomedicine. 93, 153781. 10.1016/j.phymed.2021.153781 34649212

[B120] Wang S. Fu J. L. Hao H. F. Jiao Y. N. Li P. P. Han S. Y. (2021c). Metabolic reprogramming by traditional Chinese medicine and its role in effective cancer therapy. Pharmacol. Res. 170, 105728. 10.1016/j.phrs.2021.105728 34119622

[B121] Wang Y. Hao F. Nan Y. Qu L. Na W. Jia C. (2018). PKM2 inhibitor shikonin overcomes the cisplatin resistance in bladder cancer by inducing necroptosis. Int. J. Biol. Sci. 14 (13), 1883–1891. 10.7150/ijbs.27854 30443191 PMC6231221

[B122] Wang Z. Wang N. Chen J. Shen J. (2012). Emerging glycolysis targeting and drug discovery from Chinese medicine in cancer therapy. Evid. Based. Complement. Altern. Med. 2012, 873175. 10.1155/2012/873175 PMC3403522 22844340

[B123] Wasik A. A. Lehtonen S. (2018). Glucose transporters in diabetic kidney disease-friends or foes? Front. Endocrinol. 9, 155. 10.3389/fendo.2018.00155 PMC5900043 29686650

[B124] Wei J. Wu J. Xu W. Nie H. Zhou R. Wang R. (2018b). Salvianolic acid B inhibits glycolysis in oral squamous cell carcinoma via targeting PI3K/AKT/HIF-1α signaling pathway. Cell Death Dis. 9 (6), 599. 10.1038/s41419-018-0623-9 29789538 PMC5964095

[B125] Wei J. Wu J. Xu W. Nie H. Zhou R. Wang R. (2018a). Salvianolic acid B inhibits glycolysis in oral squamous cell carcinoma via targeting PI3K/AKT/HIF-1α signaling pathway. Cell Death Dis. 9 (6), 599. 10.1038/s41419-018-0623-9 29789538 PMC5964095

[B126] Wei X. Mao T. Li S. He J. Hou X. Li H. (2019). DT-13 inhibited the proliferation of colorectal cancer via glycolytic metabolism and AMPK/mTOR signaling pathway. Phytomedicine 54, 120–131. 10.1016/j.phymed.2018.09.003 30668361

[B127] Wokolorczyk D. Gliniewicz B. Sikorski A. Zlowocka E. Masojc B. Debniak T. (2008). A range of cancers is associated with the rs6983267 marker on chromosome 8. Cancer Res. 68 (23), 9982–9986. 10.1158/0008-5472.Can-08-1838 19047180

[B128] Wu H. Cui M. Li C. Li H. Dai Y. Cui K. (2021). Kaempferol reverses aerobic glycolysis via miR-339-5p-mediated PKM alternative splicing in colon cancer cells. J. Agric. Food Chem. 69 (10), 3060–3068. 10.1021/acs.jafc.0c07640 33663206

[B129] Wu L. Cao K. Ni Z. Wang S. Li W. Liu X. (2019). Rhein reverses doxorubicin resistance in SMMC-7721 liver cancer cells by inhibiting energy metabolism and inducing mitochondrial permeability transition pore opening. Biofactors 45 (1), 85–96. 10.1002/biof.1462 30496631

[B130] Xiang Y. Guo Z. Zhu P. Chen J. Huang Y. (2019). Traditional Chinese medicine as a cancer treatment: Modern perspectives of ancient but advanced science. Cancer Med. 8 (5), 1958–1975. 10.1002/cam4.2108 30945475 PMC6536969

[B131] Xie X. Huang X. Tang H. Ye F. Yang L. Guo X. (2018). Diallyl disulfide inhibits breast cancer stem cell progression and glucose metabolism by targeting CD44/PKM2/AMPK signaling. Curr. Cancer Drug Targets 18 (6), 592–599. 10.2174/1568009617666171024165657 29110616

[B132] Xu Q. Dou C. Liu X. Yang L. Ni C. Wang J. (2018). Oviductus ranae protein hydrolysate (ORPH) inhibits the growth, metastasis and glycolysis of HCC by targeting miR-491-5p/PKM2 axis. Biomed. Pharmacother. 107, 1692–1704. 10.1016/j.biopha.2018.07.071 30257387

[B133] Yang P. Ding G. B. Liu W. Fu R. Sajid A. Li Z. (2018). Tannic acid directly targets pyruvate kinase isoenzyme M2 to attenuate colon cancer cell proliferation. Food Funct. 9 (11), 5547–5559. 10.1039/c8fo01161c 30259036

[B134] Yang Y. Liu L. Sun J. Wang S. Yang Z. Li H. (2021). Deoxypodophyllotoxin inhibits non-small cell lung cancer cell growth by reducing HIF-1α-Mediated glycolysis. Front. Oncol. 11, 629543. 10.3389/fonc.2021.629543 33732648 PMC7959795

[B135] Yao J. Man S. Dong H. Yang L. Ma L. Gao W. (2018). Combinatorial treatment of Rhizoma Paridis saponins and sorafenib overcomes the intolerance of sorafenib. J. Steroid Biochem. Mol. Biol. 183, 159–166. 10.1016/j.jsbmb.2018.06.010 29932973

[B136] Yoon Y. J. Kim Y. H. Jin Y. Chi S. W. Moon J. H. Han D. C. (2018). 2'-hydroxycinnamaldehyde inhibits cancer cell proliferation and tumor growth by targeting the pyruvate kinase M2. Cancer Lett. 434, 42–55. 10.1016/j.canlet.2018.07.015 30009856

[B137] Zhang C. Cai T. Zeng X. Cai D. Chen Y. Huang X. (2018). Astragaloside IV reverses MNNG-induced precancerous lesions of gastric carcinoma in rats: Regulation on glycolysis through miRNA-34a/LDHA pathway. Phytother. Res. 32 (7), 1364–1372. 10.1002/ptr.6070 29577459

[B138] Zhang J. Zhou J. Xiao S. (2020). Shikonin inhibits growth, invasion and glycolysis of nasopharyngeal carcinoma cells through inactivating the phosphatidylinositol 3 kinase/AKT signal pathway. Anticancer. Drugs 31 (9), 932–941. 10.1097/CAD.0000000000000920 32282369

[B139] Zhang Q. Luo P. Xia F. Tang H. Chen J. Zhang J. (2022a). Capsaicin ameliorates inflammation in a TRPV1-independent mechanism by inhibiting PKM2-LDHA-mediated Warburg effect in sepsis. Cell Chem. Biol. 29, 1248–1259.e6. 10.1016/j.chembiol.2022.06.011 35858615

[B140] Zhang T. Lin C. Wu S. Jin S. Li X. Peng Y. (2022b). ACT001 inhibits TLR4 signaling by targeting Co-receptor MD2 and attenuates neuropathic pain. Front. Immunol. 13, 873054. 10.3389/fimmu.2022.873054 35757727 PMC9218074

[B141] Zhao X. Zhu Y. Hu J. Jiang L. Li L. Jia S. (2018). Shikonin inhibits tumor growth in mice by suppressing pyruvate kinase M2-mediated aerobic glycolysis. Sci. Rep. 8 (1), 14517. 10.1038/s41598-018-31615-y 30266938 PMC6162216

[B142] Zhou L. Yu X. Li M. Gong G. Liu W. Li T. (2020). Cdh1-mediated Skp2 degradation by dioscin reprogrammes aerobic glycolysis and inhibits colorectal cancer cells growth. EBioMedicine 51, 102570. 10.1016/j.ebiom.2019.11.031 31806563 PMC7000337

